# Phylogenomic Analysis Resolves the Formerly Intractable Adaptive Diversification of the Endemic Clade of East Asian Cyprinidae (Cypriniformes)

**DOI:** 10.1371/journal.pone.0013508

**Published:** 2010-10-20

**Authors:** Wenjing Tao, Ming Zou, Xuzhen Wang, Xiaoni Gan, Richard L. Mayden, Shunping He

**Affiliations:** 1 Key Laboratory of Aquatic Biodiversity and Conservation of Chinese Academy of Sciences, Institute of Hydrobiology, Chinese Academy of Sciences, Wuhan, People's Republic of China; 2 Graduate University of Chinese Academy of Sciences, Beijing, People's Republic of China; 3 Laboratory of Integrated Genomics, Biodiversity, and Conservation, Department of Biology, Saint Louis University, Saint Louis, Missouri, United States of America; University of Zurich, Switzerland

## Abstract

Despite their great diversity and biological importance, evolutionary relationships among the endemic clade of East Asian Cyprinidae remain ambiguous. Understanding the phylogenetic history of this group involves many challenges. For instance, ecomorphological convergence may confound morphology-based phylogenetic inferences, and previous molecular phylogenetic studies based on single genes have often yielded contradictory and poorly supported trees. We assembled a comprehensive data matrix of 100 nuclear gene segments (∼ 71132 base pairs) for representative species of the endemic East Asian cyprinid fauna and recovered a robust phylogeny from this genome-wide signal supported by multiple analytical methods, including maximum parsimony, maximum likelihood and Bayesian inference. Relaxed molecular clock analyses indicated species radiations of this clade concentrated at approximately 1.9–7.6 MYA. We provide evidence that the bursts of diversification in this fauna are directly linked to major paleoenvironmental events associated with monsoon evolution occurring from late Miocene to Pliocene. Ancestral state reconstruction reveals convergent morphological characters are hypothesized to be independent products of similar selective pressures in ecosystems. Our study is the first comprehensive phylogenetic study of the enigmatic East-Asian cyprinids. The explicit molecular phylogeny provides a valuable framework for future research in genome evolution, adaptation and speciation of cyprinids.

## Introduction

With about 210 genera and 2010 species distributed across Eurasia, the East Indian Islands, Africa and North America [1], Cyprinidae is the largest family of freshwater fish in the world. The endemic clade of East Asian Cyprinidae displays a tremendous diversity of ecological and phenotypic traits, enabling them to exploit river drainages and lakes in this area. As was the case for the well-studied cichlid model [2], this clade is an ideal and attractive model system to study rapid radiations, and evolutionary adaptations in freshwater fishes.

Endemic East Asian cyprinids exhibit reproductive diversity between riverine and lacustrine species. Riverine species produce pelagic eggs, which require the stimulation of flowing water for development. Lacustrine species lay viscid eggs and spawn in still water. Several riverine species, particularly *Hypophthalmichthys*, have been introduced into river and lake ecosystems around the world and are, in most instances, highly problematic nuisance species that degrade aquatic ecosystems, leading to threats to conservation status, and even extinction of many native species. The radiation of this clade must have involved attributes in all species that has enhanced their success beyond their native range. Thus, a robust estimate of phylogeny and divergence time of this group is of great importance and may facilitate the elucidation of important factors for the development of effective control methods in freshwater ecosystems where they exist as exotics.

**Figure 1:**
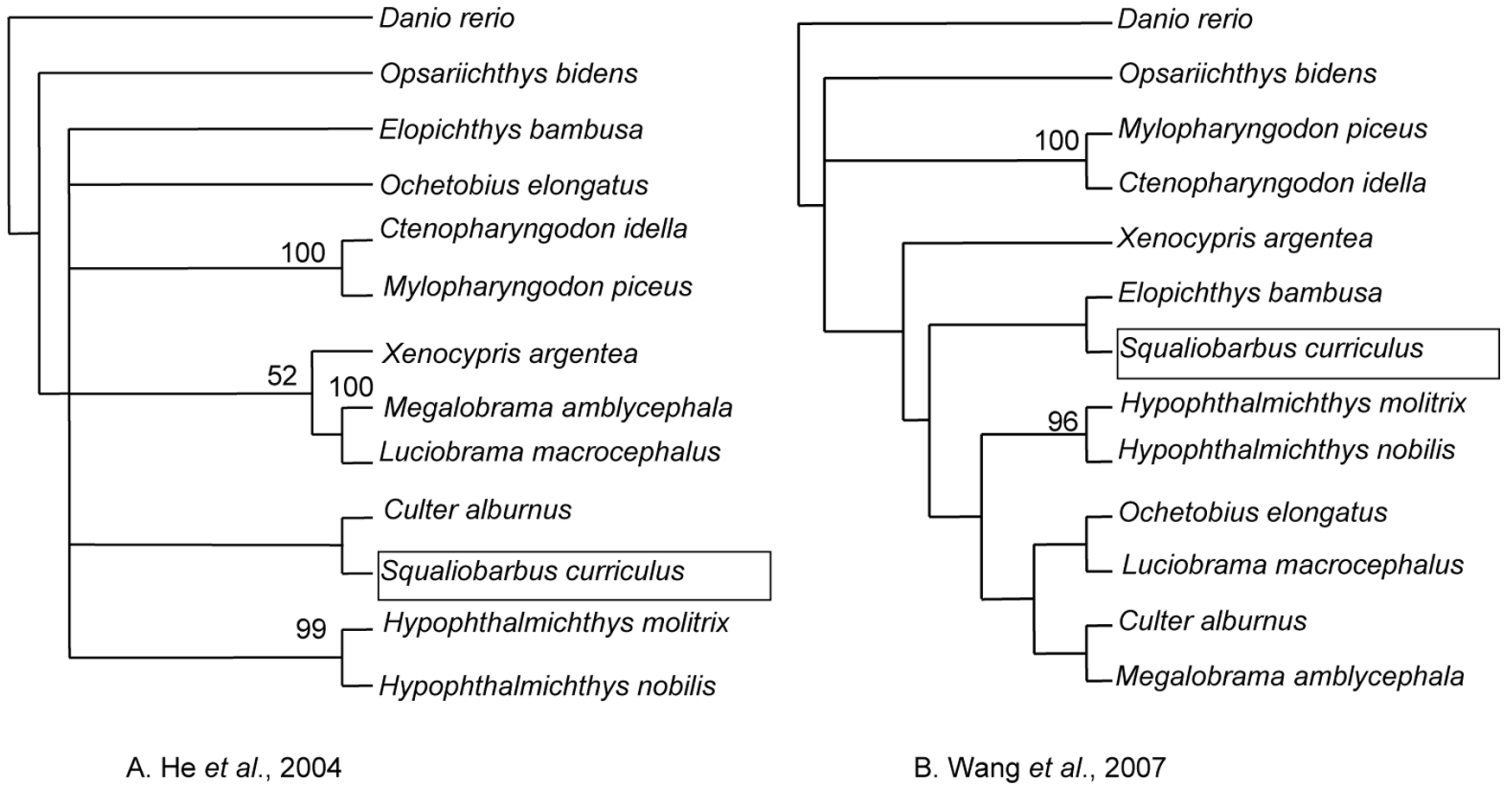
Previously published phylogenetic hypotheses for the East Asian clade of Cyprinidae. Previous studies resulted in limited resolution of relationships within the endemic East Asian clade of cyprinids. The placement of highlighted *Squaliobarbus* and relationships of the species *Mylopharyngodon piceus*, *Ctenopharyngodon idella*, *Elopichthys bambusa*, *Ochetobius elongatus*, and *Luciobrama macrocephalus* are far from being understood.

Previous studies attempting to resolve the relationships among East Asian cyprinid species strongly support their monophyly and placement in Cyprinidae [3]–[7]. Nevertheless, inter-relationships of species within this clade have remained largely intractable and unresolved (Figure 1). As an example, molecular analysis of mitochondrial cytochrome b sequences (cytb) placed *Culter alburnus* sister to *Squaliobarbus curriculus* [5] (Figure 1A), whereas sequence variability of the nuclear recombination activating gene 2 sequences (RAG2) placed *Culter alburnus* more closely related to *Megalobrama amblycephala* [3] (Figure 1B).

Previous efforts to reconstruct phylogenetic relationships of endemic East Asian cyprinids have been problematic and of limited success with the use of data yielding short internodes derived from morphological characters [8], mitochondrial genes [5], [6],and two nuclear genes [3], [7]. Character anagenesis in a lineage is often positively correlated with time since isolation [9], [10]. Thus, species radiation associated with cladogenesis over short intervals offers a limited opportunity for the anagenesis of apomorphic traits to evolve and accumulate in common ancestral species. They are thus predicted to result short internodes and weak support, as measured by jackknife and bootstrap resampling [10]. Unless the characters that did change during this short interval are examined there will be no resolution to this problem. Secondly, evolutionary rates of mitochondrial sequences often differ greatly among different lineages [11]–[13]. Thus phylogenetic analyses of endemic cyprinids in East Asia based on cytb [5] as well as ribosome gene (16 s) [14] may be less likely to represent the species tree because of inherent attributes such as hybridization or introgression, independence of contiguous genes, and maternally inherited genomes [15]. Thirdly, phylogenetic hypotheses based on a single nuclear gene are even less well-resolved because of three sources of variation between genes: stochastic nature, lineage sorting and sensitivity to paralogy [16], [17].

Phylogenomics, an evolutionary and phylogenetic approach to comparative genomics, has given rise to a wealth of genomic data and has successfully resolved some long-standing phylogenetic problems [18]–[20]. With more and more genomes being sequenced, multigene phylogenies or so-called phylogenomics has opened up a new era for elucidating previously intractable or controversial evolutionary relationships. Phylogenomic analysis can make use of existing database (for example, protein sequences sampled from the Swiss-Prot and GenBank), PCR-targeted single-copy genes [21], [22], ESTs [23], [24] and whole genomes [25]. These methods of data generation, owing to the developing genomic-level sequencing techniques have been used to address controversial phylogenetic problems of non-model species. The completion of the genome of *Danio rerio* has made it and other species of Cyprinidae increasingly attractive as a “model system of diversity” for biological studies at the genomic level, enabling inferences to be made on an impressive array of comparative and evolutionary questions. Herein, we made use of the phylogenomic approach and available resources via *D. rerio* to sample multiple nuclear genes and incorporated additional data from Genbank to reconstruct the long-standing problematic phylogenetic relationships of the endemic cyprinids in East Asia. The following objectives were addressed: (1) elucidate the phylogenetic relationships among East Asian cyprinids, (2) evaluate the performance of nuclear loci for phylogenetic and evolutionary studies, (3) test if the divergence events of *Megalobrama amblycephala* and *Culter alburnus* correlate with evolution of the East Asian winter monsoon [26], and (4) gain a better understanding of rapid diversification and adaptive evolution of the endemic East Asian cyprinids through ancestral-state reconstructions.

## Materials and Methods

### Sampling single-copy genes

The decisive role of orthologues in avoidance of erroneous speculations of species tree has been highlighted in many cases [17], [27], [28]. The genes we used in this phylogenomic analysis were thus carefully selected to avoid fundamental errors in homology. We implemented a bioinformatics pipeline for mining of single-copy genes. Whole genomic sequences of *D. rerio* were retrieved from the Ensembl database for gene screening [29]. We extracted the protein sequence and conducted extensive searches against the genomic sequences in all six possible reading frames using t-blastn at the e-values of 10^−1^. To obtain single-copy genes for future analyses, only protein-coding sequences with both similarity (S) and coverage (C) of less than 30% within-genome sequence comparisons were retained. That is to say, only sequences that have no duplicates over 30% similar to themselves in *D. rerio* genome were selected for further analyses. We then performed t-blastx searches using sequences of these candidate genes against Genbank to obtain orthologues from fugu (*Fugu rubripes*) and medaka (*Oryzias latipes*). We selected genes with the reported criteria [15]: not only were these selected genes conservative enough for primer design, but they were also parsimony informative for the resolution of a variable range of intractable phylogenetic problems.

### Species sampling and data assembly

For this study, multigene sequences were determined for 13 species of Cyprinidae, including representative East Asian cyprinids (one individual per species) and *D. rerio* (Table 1). Total DNA was extracted from muscle or fin tissues using phenol/chloroform extraction procedure [30]. Primers for PCR of all 100 genes are listed (Appendix S1). PCR amplification was carried out for 35 cycles, under the following conditions: an initial denaturation at 94°C for 5 min, denaturation at 94°C for 30 s, annealing at 48–56°C for 30 s–50 s, extension at 72°C for 30–120 s, and a final extension of 8 min at 72°C. To provide a check for orthology, amplified products are with a single prominent band. Amplified DNA fragments were fractionated by electrophoresis through 1.0% low-melting agarose gels. Products of expected size were sequenced either directly or after cloning into PMD18-T vectors (Takara). Because of amplification difficulties, some data were missing and partial sequences were present in some species. Missing data or incomplete sequences did not, however, affect the inferred phylogeny because the dataset in our study provided sufficient information, consistent with previous empirical studies [22], [31], [32].

**Table 1:** Taxa and sample location of species included in this study.

Subfamily	Species	Sample location	No. of sites (bp)
Leuciscinae	*Mylopharyngodon piceus*	Wuhan, Hubei Prov.	57624
	*Ctenopharyngodon idella*	Wuhan, Hubei Prov.	62541
	*Squaliobarbus curriculus*	Taoyuan, Hunan Prov.	57357
	*Ochetobius elongatus*	Taoyuan, Hunan Prov.	56865
	*Luciobrama macrocephalus*	Taoyuan, Hunan Prov.	48501
	*Elopichthys bambusa*	Taoyuan, Hunan Prov.	55421
Hypophthalmichthyinae	*Hypophthalmichthys molitrix*	Wuhan, Hubei Prov.	61271
	*Hypophthalmichthys nobilis*	Wuhan, Hubei Prov.	62371
Xenocyprinae	*Xenocypris argentea*	Taoyuan, Hunan Prov.	53348
Cultrinae	*Culter alburnus*	Taoyuan, Hunan Prov.	58803
	*Megalobrama amblycephala*	Wuhan, Hubei Prov.	60738
Danioninae	*Opsariichthys bidens*	Taoyuan, Hunan Prov.	55835
	*Danio rerio*	--------	56378

Note: genome sequences of *Danio rerio* were retrieved from GenBank or Ensembl database. Species of Cyprinidae grouped on the basis of traditional subfamily classifications. One individual per species was sampled.

Using experimentally amplified sequences as queries, we performed t-blastx searches against the database of GenBank in NCBI (http://www.ncbi.nlm.nih.gov/) to confirm that there was only one significant hit for each genetic marker, thus avoiding potentially paralogous comparisons [33]. Multiple alignments were carried out using default parameters in Bioedit (Biological sequence alignment editor V5.0.9, http://www.mbio.ncsu.edu/Bioedit/bioedit.html). Frame shifts or indels detected in exon and intron were manually excluded. Exons were easily aligned; however, non-coding sequences required greater effort in alignment because these regions had higher variability and repeated stretches of monomers. Alignments of individual genes are available from the authors upon request. We chose to exclude regions of each gene that showed evidence of high levels of saturation by multiple substitutions, and poor sequencing quality from phylogenetic analyses. All sequences amplified in this study were deposited in GenBank (accession numbers GU217798 to GU218392, and GU218394 to GU218691; Appendix S2). We evaluated alignment statistics of individual gene, including length, percentage of exon, ratio of variable and parsimony informative characters, average within-group p-distance, and average base composition using MEGA4 [34] and Seqstate [35].

### Sequence and phylogentic analyses

The aligned sequences were concatenated using a custom Perl script, and was used for all phylogenetic inference. Heterogeneity of the nucleotide base composition was tested using Chi-square test in PAUP* version 4.0b10 [36]. Parameters such as base frequencies, numbers of substitution types, proportion of invariable sites, and Gamma distribution shape were optimized using Modeltest3.7 [37] with the Akaike Information Criterion (AIC). We performed the heuristic searches option with tree bisection-reconnection (TBR) branch-swapping under Maximum Parsimony (MP). All characters were treated as equally weighted. Node support values in MP analyses were assessed using non-parametric bootstrapping for 1000 pseudo-replicates (10 random taxon addition sequence replicates per pseudo-replicate). We used PhyML [38] to determine Maximum likelihood (ML) tree with the optimal model. Robustness of lineages was tested by bootstrap analyses based on 1000 rounds of bootstrap resampling. Bayesian inference (BI) was conducted using MrBayes v3.1.2 [39], in which four independent runs of Metropolis-coupled chains (MCMC) with 2000000 generations to estimate the posterior probability distribution (sampling one tree per 1000 replicates for each run). After discarding the first 1000 trees as burn-in with non-stationary log likelihood values, 50% majority-rule consensus trees were estimated for the remaining trees. Stability of nodes was estimated using posterior probabilities (PP). Ancestral state reconstruction was also performed using MrBayes. To compare alternative topologies obtained from previous studies [3], [4] with the combined datasets, site-wise log-likelihoods for candidate trees were calculated using PAUP*4.0b and used as inputs into the CONSEL program package [40]. The p-value was calculated using Approximately Unbiased (AU) test, Bootstrap Probability (BP) test, Kishino-Hasegawa (KH) test, Shimodaira-Hasegawa (SH) test and Weighted Shimodaira-Hasegawa (WSH) test.

We employed variable length bootstrap analysis to investigate the minimum length required to obtain robust phylogenetic inference for this group. In this analysis, bootstrap support of resampled characters was estimated at variable sequence lengths [14], [41]. All bootstrap searches were performed using MP analyses with PAUP* version 4.0b10 and the number of resampled bases extended from 2000 to 500000 characters to generate bootstrap pseudomatrix.

For comparison, we evaluated the relative contribution or effect of each gene using a decay index or Bremer support index in TreeRot3 [42]. Partitioned Bremer Support (PBS) was calculated following the method of Baker & DeSalle [43]. Individual PBS scores can be positive, negative and zero. Positive PBS values indicate that a given dataset increases support for particular node whereas negative values show that data partition provides net negative support for the given node. A PBS value of zero suggests that the given data partition at that node has an indifferent relationship. Thus, the larger the PBS values for a node of interest, the greater the relative effectiveness of that genetic marker in resolving and supporting that node. The sum of PBS values of the different data partitions for any given node will always be equal to the decay index for the node of the inferred tree.

Phylogenetic studies with relatively few taxa have a major advantage in terms of exploring a variety of analytical methods [22], [44] and all possible phylogenetic reconstructions. Several discussions have raised legitimate arguments against the naturalness of data partitioning and choice of model selection [45]–[48]. To examine the potential systematic errors caused by model misspecification and improper data partitioning strategies, we applied a series of data partitioning strategies, and homogenous versus mixed models (parameters were unlinked across partitions). We then evaluated the relative merits of competing data partitioning strategies and alternative models by Bayes factors. The analysis does not require the assumption of any asymptotic property and hierarchically nested hypotheses but it provides a rigorous basis for model testing or data partitioning in terms of probability [49]. We approximated the Bayes factor as the marginal likelihood (the ratio of the harmonic mean of likelihoods) of Markov Chain Monte Carlo samples [50]. We calculated twice the natural logarithm of the Bayes factors for alternative partitioning strategies, and determined the result using the criteria provided by Kass and Ratery [51]: the null hypothesis is preferred if 2lnBF <0, which provides evidence in favor of model 0; on the other hand, when 2lnBF > 10 the null hypothesis is rejected. The partitioning strategies were as follows: (1) equal length partitioning (dividing the concatenated data to 7 partitions of equal sequence length), (2) partitioning by exon and intron (exon + intron), (3) partitioning by codon positions and intron (1st codon position + 2nd codon position + 3rd codon position + intron), (4) all data combined in one single partition (one partition), and (5) partitioning by genes (100 gene partitions). Using these varied partitions, results of analyses were compared, to test which was the most suitable one for improving phylogenetic inference. To eliminate model misspecification, we also used Bayes factors with the above-mentioned criteria to evaluate the relative merits of homogeneous models and mixed models.

To estimate divergence times, likelihood ratio test was performed using PAUP*4.0b to obtain the likelihood scores and investigate whether a global clock fit the combined dataset. Divergence times were estimated using Multidivtime [52]–[54]. We chose the default F84 model, the most complicated model with four discrete categories for the Γ distribution of rate in Multidivtime. Divergence time algorithms require calibration for at least one internal node. Minimum age constraints were determined using fossil records of extant cyprinids in China from the Pliocene (5.33–1.81 MYA). We chose the recent fossil-based time constraints assignable to *Mylopharyngodon piceus* and *Ctenopharyngodon idella* with a minimum age of 1.81 MYA [55].

## Results

### Characteristics of potential markers

Our definition of a single-copy gene required no duplicates that were more than 30% similar in *D. rerio* genome. The bioinformatics approach yielded a total of 1042 candidate single-copy genes that were most likely free of the paralogy problem in *D. rerio*. Among these genes, we found 158 single-copy genes with exon lengths > 800 bp, 204 genes with exon lengths from 700 bp to 800 bp, 279 genes with exon lengths from 600 bp to 700 bp, and 401 genes with exon lengths from 500 bp to 600 bp ( See Appendix S3 for gene accession numbers). The actual number of candidate single-copy genes depended, however, on *a priori* search parameters.

Randomly picked gene fragments from candidate list combined with previously developed nuclear markers were used to investigate the inter-relationships among the endemic clade of East Asian Cyprinidae. The final alignment included 100 nuclear genes into a data matrix of 71132 bp, with exons accounting for 71.8% of the total sequence. These selected genes are distributed throughout *D. rerio* chromosomes and represent a genome-wide sampling of molecular markers. Of these sites, 12433 bp were variable (21.5%), and 3156 were parsimony informative (4.44%) (Appendix S4). Mean base composition was found to be fairly uniform among all taxa analyzed (A = 25.9%, C = 26.1%, G = 23.8%, T = 24.3%). Chi-square tests of homogeneous base frequencies among all partition strategies were listed in Appendix S5. The overall transition to transversion ratio of concatenated data was 1.847. The Kimura two-parameter pairwise distance showed low levels of genetic divergences among the endemic East Asian cyprinids (Table 2).

**Table 2:** Pairwise Kimura two-parameter distances between species.

	A	B	C	D	E	F	G	H	I	J	K	L
B	0.0185											
C	0.0246	0.0270										
D	0.0235	0.0269	0.0151									
E	0.0194	0.0237	0.0268	0.0281								
F	0.0275	0.0304	0.0288	0.0306	0.0307							
G	0.0221	0.0262	0.0304	0.0313	0.0279	0.0339						
H	0.0282	0.0330	0.0262	0.0329	0.0329	0.0293	0.0349					
I	0.0276	0.0310	0.0330	0.0311	0.0298	0.0200	0.0336	0.0312				
J	0.0397	0.0453	0.0455	0.0484	0.0457	0.0500	0.0500	0.0491	0.0473			
K	0.0208	0.0252	0.0286	0.0301	0.0256	0.0297	0.0278	0.0339	0.0301	0.0442		
L	0.0219	0.0286	0.0325	0.0330	0.0267	0.0312	0.0324	0.0370	0.0324	0.0480	0.0254	
M	0.1066	0.1083	0.1110	0.1131	0.1058	0.1116	0.1136	0.1118	0.1076	0.1078	0.1102	0.1098

Note: A-*Mylopharyngodon piceus*; B**-** *Ctenopharyngodon idella;* C-*Hypophthalmichthys molitrix*; D-*Hypophthalmichthys nobilis*; E- *Squaliobarbus curriculus;* F-*Megalobrama amblycephala*; G- *Elopichthys bambusa*; H- *Xenocypris argentea;* I- *Culter alburnus*; J- *Opsariichthys bidens;* K- *Ochetobius elongatus*; L- *Luciobrama macrocephalus*; M-*Danio rerio*.

### Phylogenetic inferences from the 100 concatenated genes

**Figure 2:**
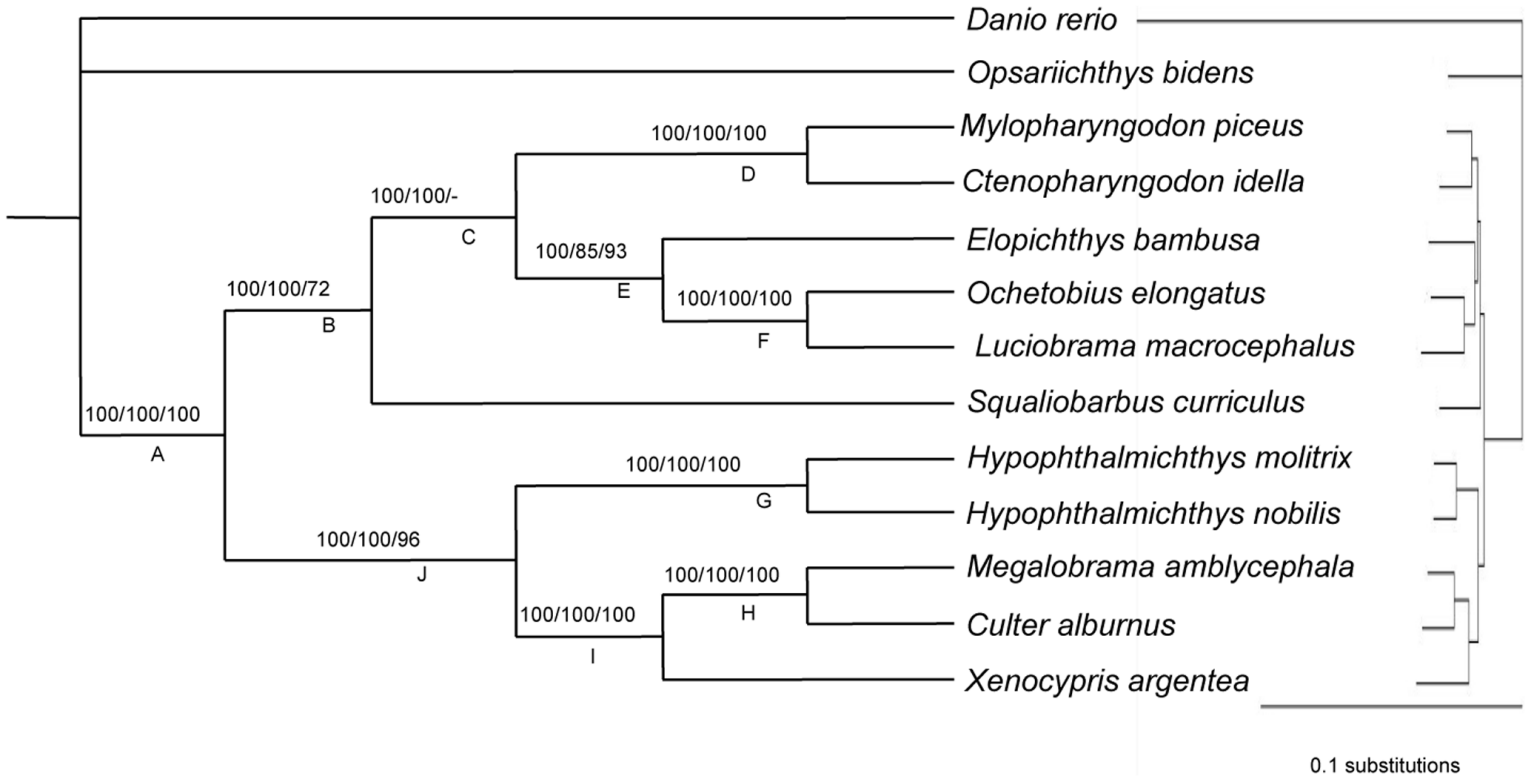
Phylogenetic relationships of the endemic East Asian cyprinids inferred from the concatenated sequences. Numbers above branches represent posterior probability of BI, and the bootstrap support of ML and MP, respectively. And on the right side is the phylogram. The symbol “-” indicates the branch was unresolved (bootstrap value less than 70%).

ML, MP and BI analyses yielded one fully-resolved topology with all internal nodes receiving nearly 100% bootstrap support and posterior probabilities (Figure 2). The congruent tree recovered two major clades with clear relationships among the endemic cyprinids. The first clade included *My. piceus* being sister to *Ct. idella*, which branched with the strongly supported assemblage wherein *Elopichthys bambusa* formed the sister group to *Luciobrama macrocephalus* plus *Ochetobius elongatus. Squaliobarbus curriculus*, which displayed variable relationships in previously reported phylogenetic hypotheses (Figure 1), is now consistently supported as the basal group to all other members of this clade. The second clade could be subdivided into two strongly supported subgroups. The first subgroup included *Hypophthalmichthys nobilis* sister to *Hypophthalmichthys molitrix*, a clade that formed the sister-group to the remaining members of the subgroup. Among remaining members, *Xenocypris argentea* branched with *M. amblycepha* plus *C. alburnus*. According to alternative topology test analyses, the topology derived from the concatenated matrix was favored and all other competing phylogenetic hypotheses were rejected by significantly lower probabilities (Appendix S6).

**Figure 3:**
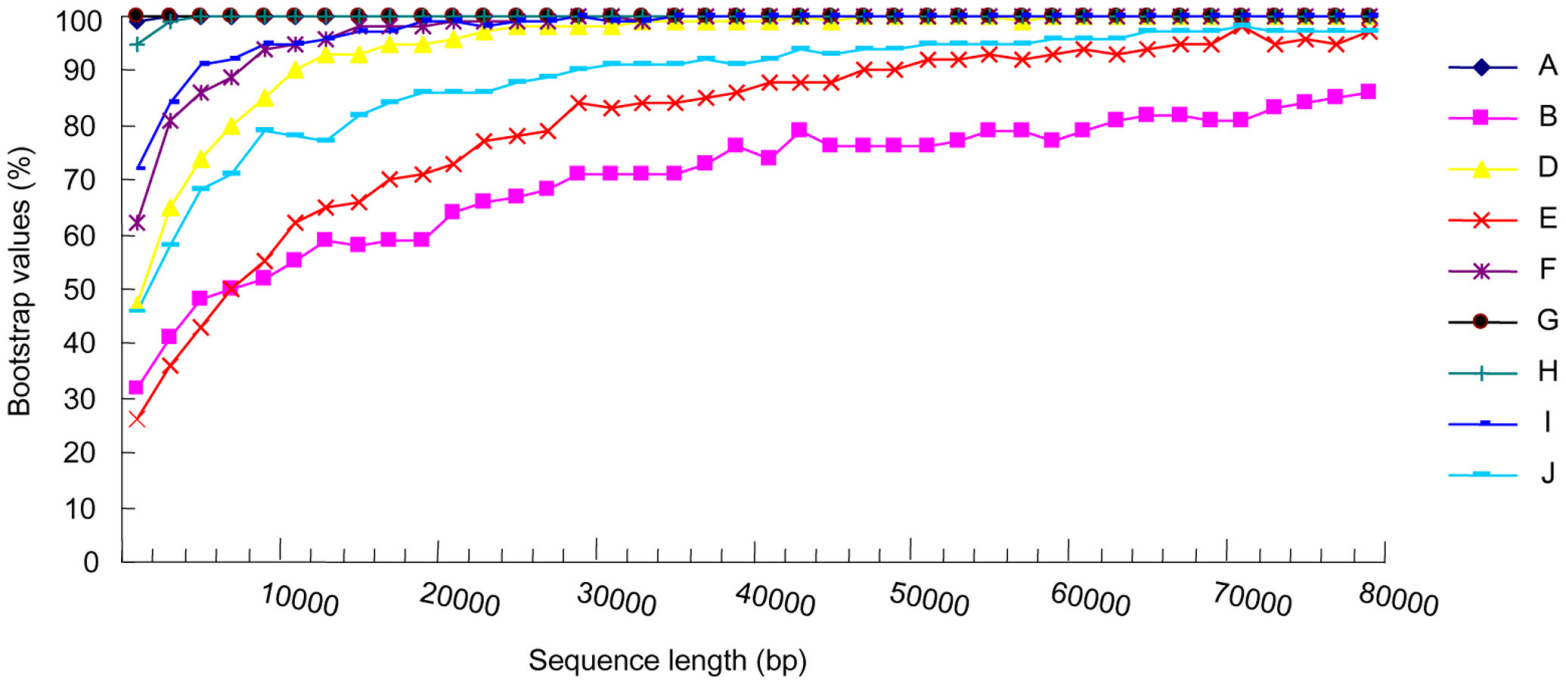
Plots of variable length variation of concatenated dataset and bootstrap values attained for nodes in the inferred simultaneous tree (**Figure 2**) using variable length bootstrap. Node C was not included due to moderate bootstrap values in the combined analyses.

To assess how large of the data set might be needed to resolve a phylogeny, we explored the relationships between the number of nucleotide sites and phylogenetic resolution. The variable length bootstrap curves clearly demonstrated that improvement of bootstrap values was significant for each node, with the resampling of more sites (Figure 3). Nodes B, D, and E achieved at least a 50% bootstrap support increase with the resampling sites up to 70000 bp. Improvement was significant for all nodes when the length of resampling sites ranged from 2000 bp to 30000 bp. All nodes except node C received 100% bootstrap support when resampling sites was 500000 bp (not shown). Little improvements of bootstrap values were, however, found for node C, even with the resampling sites exceeding 500000 bp (not shown).

Partitioned Bremer support (PBS) across the 100 nuclear markers in combined Bayesian analyses was used to evaluate the contribution of individual marker to the overall support for the tree (summarized in Appendix S7). This index demonstrated that the marker of interphotoreceptor retinoid-binding protein (PBS value: 50) contributed most to the concatenated analysis, while the zinc finger BTB domain (PBS value: −46) showed the greatest conflict at most of the nodes. Most genes were incongruent with the consensus tree at least at one node, probably because of a lack of resolution in the individual analysis. Despite strong support for each node, it showed a more even mixture of positive and negative PBS scores, which may indicate the positive or negative contribution of individual gene to the combined tree.

### Evaluation of performance of data partitioning, model selection

We examined the performance of different partitioning strategies and model selections (Tables 3 and 4). In comparison with Bayes factors between different partitioning strategies, partitioning by exon and intron outperformed all other partitioning schemes. Values of Bayes factor became remarkably more negative when partitioning with sequence length and genes (2lnBF = 2749.56 and 7412.24, respectively), which again emphasized the importance of partitioning based on biological relevance of sequence structure and function to isolate conflicting characters and improve model fit [44]. The relative merits of competing models evaluated by Bayes factors indicated that all mixed models significantly improved model fit compared to the homogeneous models, especially the JC model (Table 4). Nevertheless, both alternative partitioning schemes and analyzed models yielded the same topology with 100% posterior probabilities at the internal nodes as shown in Figure 2. This, on the other hand, strongly suggests that phylogenetic reconstruction of this rapidly evolving clade is not biased by model misspecification.

**Table 3:** Summary of alternative partitioning strategies when Bayesian analyses were conducted.

Paired data partitioning		Harmonic means		Bayes factors	
Model 0	Model 1	Model 0	Model 1	lnBF	2lnBF
Exon + intron	1 partition (combined dataset)	−193798.37	−195500.09	1701.72	3403.44
Exon + intron	100 partitions (genes)	−193798.37	−197504.49	3706.12	7412.24
Exon + intron	4 partitions (each codon + intron)	−193798.37	−194399.00	600.63	1201.26
Exon + intron	7 partitions (equal length)	−193798.37	−195173.15	1374.78	2749.56

Note: Bayes factor comparisons preferred partitioning strategy by exon and intron.

**Table 4:** Summary of alternative models used when Bayesian analyses were conducted.

Paired models		Harmonic means		Bayes factors	
Model 0	Model 1	Model 0	Model 1	lnBF	2lnBF
GTR+I+G	HKY+I+G	−195500.09	−195549.60	49.51	99.02
GTR+I+G	GTR+I	−195500.09	−199297.96	3797.87	7595.74
GTR+I+G	GTR+G	−195500.09	−198285.54	2785.45	5570.9
GTR+I+G	GTR	−195500.09	−198299.60	2799.51	5599.02
HKY+I+G	HKY+I	−195549.60	−198337.60	2788	5576
HKY+I+G	HKY+G	−195549.60	−195552.34	2.74	5.48
HKY	JC	−198337.81	−200525.49	2187.68	4375.36

Note: Bayes factor comparisons indicated that mixed models significantly outperformed homogeneous models.

### Divergence time estimates between lineages

**Figure 4:**
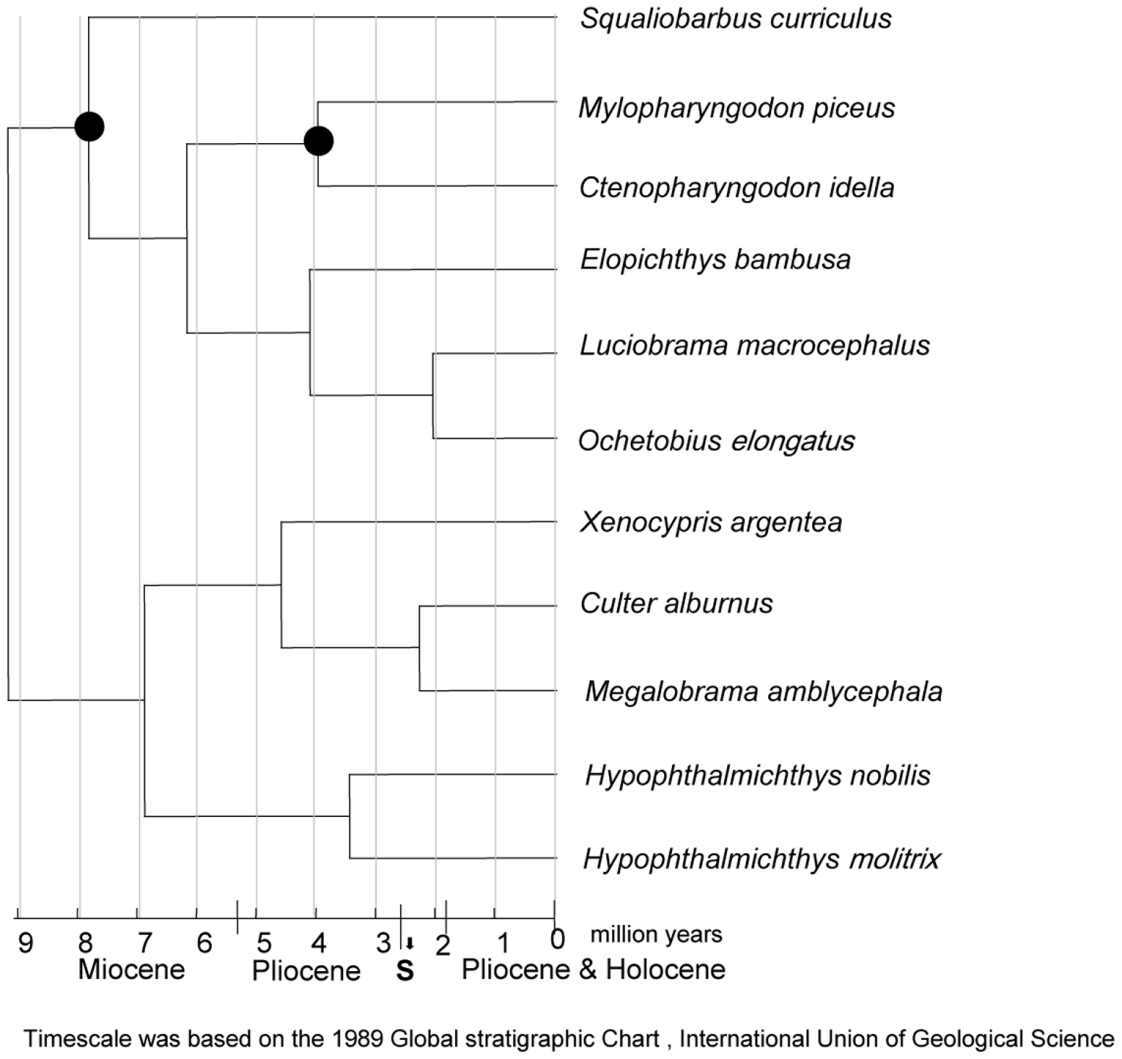
Molecular dating of the East Asian groups of cyprinids. The fossil-based constraints are indicated with black dots. Branch lengths are proportional to divergence times (in million of years). The East Asian cyprinids originated mostly in Piocene but some occurred more recently. “S” indicates possible weakening of the Indian and East Asian summer monsoons and continued strengthening of the East Asian winter monsoon, an important time of climatic change and habitat modification that existed during the evolution of the lacustrine species.

A significant difference (P<0.001) between the log likelihood values of clock-like versus non-clock-like behavior justified the use of the relaxed molecular clock model for the clade of East Asian Cyprinidae. Our results indicated that the endemic East Asian cyprinids diverged from other cyprinids ∼9 MYA (Figure 4). Of the four famous carp species in China, *My. piceus* and *Ct. idella* diverged from their most recent common ancestor ∼3.95 MYA, and the divergence between *H. molitrix* and *H. nobilis* occurred ∼ 3.41 MYA. The crown radiation of lacustrine species began ∼2.27 MYA and probably experienced effects from possible weakening of Indian and East Asian summer monsoon and continued strengthening of East Asian winter monsoon [26].

## Discussion

### Information of molecular markers

The Cypriniformes are the most diverse order of freshwater fishes in the world [56], [57]. Found on nearly every continent, these fishes are an essential protein source for many societies, are highly valued in recreational fisheries, and constitute a major component of the tropical fish trade and financial market. Cyprinids also serve as a critically important group for primary scientific investigation on a wide range of topics including evolution, biogeography, speciation, evo-devo with a vertebrate model organism *D. rerio*. Significant advances in understanding the evolutionary relationships of major cyprinid lineages have emerged recently through an international effort focused on this group, resulting in much needed phylogenetic hypotheses of major groups and species-level phylogenies [5], [56], [58]. While these studies have supported the monophyly of the endemic East Asian clade, the unavailability of appropriate genetic markers has prevented researchers from inferring species relationships and rates of anagenesis.

Based on the aforementioned principles, we searched the genome of the *D. rerio* for multiple single-copy nuclear genes. This required compliance with initial criteria for measuring evolutionary change and resolving phylogenetic relationships in this clade with minimal genetic anagenesis in ancestral lineages. Although the actual numbers of single-copy genes can change with different input parameter values, the bioinformatics pipeline implemented in this study resulted in a large set of important candidate single-copy genes useful for inferring both cladogenesis (phylogeny) and anagenesis (lineage divergence) for this enigmatic group of species. The significant increase in the number of candidate single-copy genes derived in this study is especially important for this and other groups of polyploidy fishes, in which available nuclear markers are absent or exceedingly rare. This bioinformatics approach could be applied to other groups of organisms, in order to develop more nuclear markers as long as there is information available on at least one complete genome. The identification of high-quality and easy-to-use single-copy nuclear markers will greatly facilitate the reconstruction of the tree of life. Our results also indicate the existence of many single-copy genes in cyprinids, which supports previous hypotheses that many duplicated genes are secondarily lost through lineage of diversification after a teleost-specific third round whole genome duplication [59], [60], probably due to dosage compensation [61].

Historical attempts to resolve the relationships of this endemic clade of East Asian cyprinids have encountered numerous difficulties mostly associated with resolving relationships with the limitations of traditional genetic markers. This study shows that a phylogenetic analysis with genome-wide data may be most helpful for understanding the evolutionary relationships of rapid speciation, featured by short branches.

### Convergent trait evolution and shared ancestral polymorphism in East Asian cyprinids

**Figure 5:**
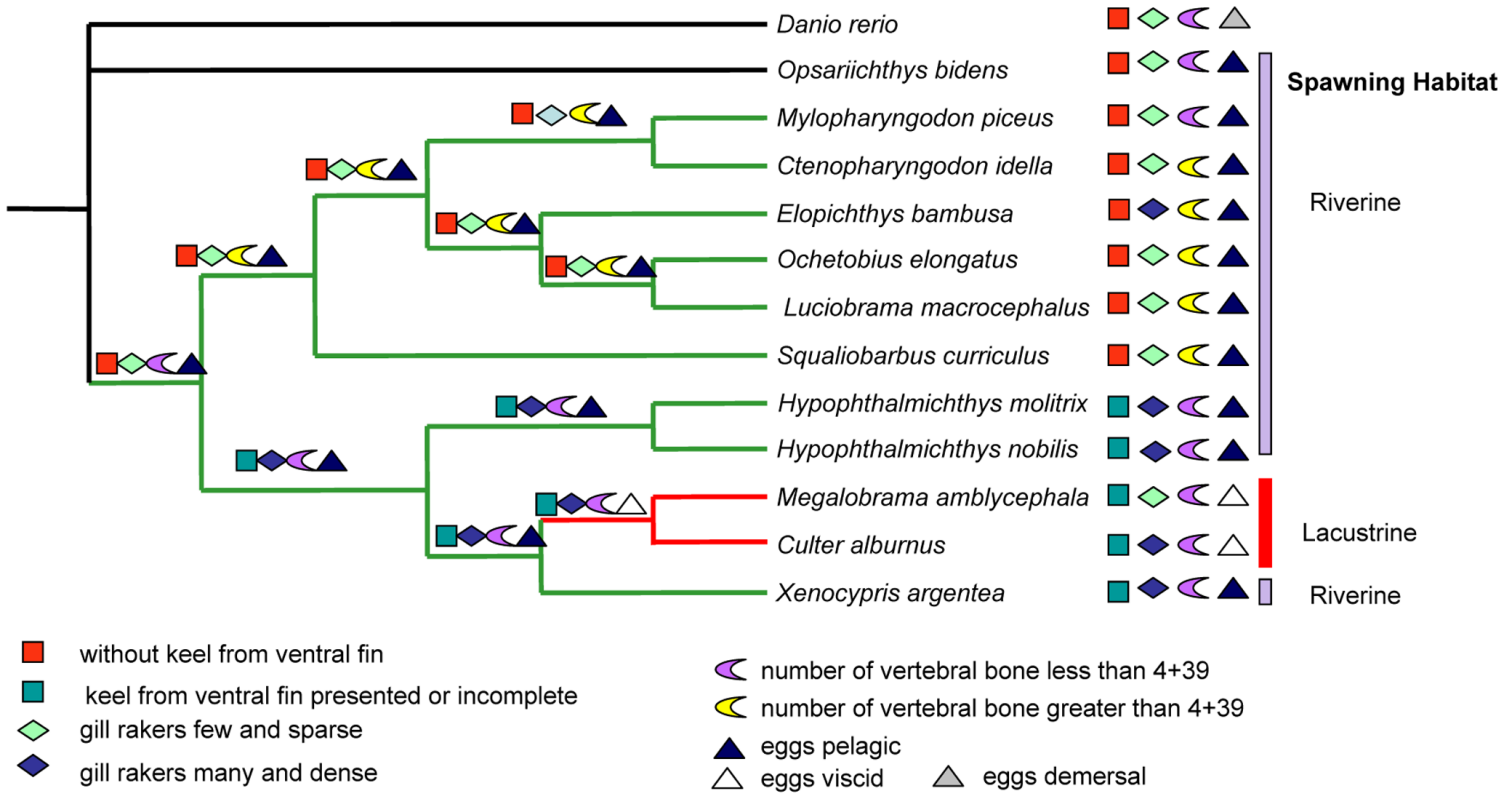
Ancestral state reconstruction for four important traits based on the obtained phylogenetic tree. The traits including the development of a ventral morphological keel from modified scales (absent/present and size), number of vertebrae, types of eggs laid (pelagic or viscid), and gill rakers morphological differences. The ancestral-state reconstruction was performed using MrBayes (2000000 generations).

Adaptive radiation is the differentiation of a single ancestor into an array of species that differ in traits to inhabit a variety of environments [62]. The extant East Asian cyprinids evolved from a single ancestor into an array of species [63] from late Miocene to the Pliocene (Figure 4). East Asian cyprinid species demonstrate repeated co-evolution of coloration, feeding morphology, and behavior in parallel [8], [63], [64]. Adaptive phenotypic differences including the development of a ventral morphological keel from modified scales (absent/present and size), number of vertebrae, types of eggs laid (pelagic or viscid), and morphological differences in gill rakers are all important sources of morphological variation involved in traditional classification and suspected to be shaped by ecological and sexual selection [8], [64]. Using MrBayes, these important morphological characters, which are reflective of the life histories and ecologies of particular species, have been used to calculate ancestral state conditions (Figure 5). Traditional classification of cyprinids based on number of vertebrae and the development of a ventral morphological keel from modified scales (absent/present and size) is not completely supported by our ancestral state reconstruction analysis, which favors an independent evolution of these morphological characters in cyprinids. Identical characters in different lineages of East Asian cyprinids may represent adaptive convergence.

The content of carotinoid and yolk differs significantly between pelagic and viscid eggs. The breeding habitat of species producing viscid eggs is subject to risks of anoxia, a physiological situation which would favor the production of more carotinoid. Species with viscid egg tend to have denser yolk to permit greater developmental differentiation at hatching. Optimization of the above adaptive ecological and morphological traits together with evidence from our molecular dating of speciation events, suggests both an ancestral state of riverine species and the later originations of the lacustrine species of this clade. Interestingly, the estimated age of the diversification of the lacustrine species overlaps significantly with and is likely to have been driven by orogenic movements and monsoon changes occurring that time. These factors include increased variability and possible weakening of the Indian and East Asian summer monsoons and intensified central Asian aridity since about 2.6 MYA [26]. The evolution of adaptive breeding habit of lacustrine species may be attributable to this arid climate change, providing insight into the mechanisms that might have been responsible for bursts of lacustrine species diversification at that time. This study confirmed a sister relationship of the coexisting filter feeders *H. molitrix* and *H. nobilis*, both of which are considered to be exotics in many countries. These species exhibit different feeding morphologies and diet composition. As is the case for scale-eating cichlids of Lake Tanganyika, “exploitative mutualism” [65] would be a key factor in the stable coexistence of these cyprinids, which occupy the same niches. Our study also reveals that *Hypophthalmichthys* include species of the highly specialized filtering apparatus of gill rakers as compared with those of other closely-related endemic East-Asian cyprinids. Future understanding of the genetic basis of this most striking characteristic may help to control these exotics.

The endemic clade of East Asian Cyprinidae has survived over great lengths of time and rapidly occupied rivers and lakes across China, developing morphological, ecological and behavioral adaptive traits in response to the unique conditions of these aquatic systems. Evolutionary processes associated with the radiation of these endemic species are hypothesized to be an example of how adaptive radiation occurred over a geologically transient environment. This clade provides an opportunity for the empirical study of adaptive evolution, as was the case for a number of other organisms, such as the house finch (*Carpodacus mexicanus*) [66], the Hawaiian silverswords [67], and the cichlids [68]. The phylogenetic relationships proposed in this paper will provide a better understanding of causes, patterns and dynamics of the relatively rapid diversification within the East Asian cyprinids. Numerous newly-developed nuclear genetic markers, as well as the resolved phylogeny, provide a valuable evolutionary framework for further research on the diversification, and taxonomic relationships at multiple levels of Cyprinidae. Further investigations into this model group of fishes should, however, be undertaken particularly with respect to their speciation and co-existence as separate lineages in unique ecological system in East Asia.

## Supporting Information

Appendix S1 Primers for PCR amplification of the sampled genes in present study.
(0.12 MB DOC)

Appendix S2 A list of Genbank accession numbers of the sequences of nuclear genes sampled in this study.
(0.28 MB DOC)

Appendix S3 Ensembl gene accession numbers of predicted single-copy nuclear genes extracted from genome sequences of Danio rerio.
(0.06 MB DOC)

Appendix S4 The detailed information for each of the 100 loci sampled in the present study.
(0.29 MB DOC)

Appendix S5 Chi-square tests of homogeneous base frequencies among all partition strategies.
(0.05 MB DOC)

Appendix S6 Statistical comparisons of alternative topologies, including the combined dataset and previous evolutionary hypothesis using approximately AU test, SH test, KH test and WKH test.
(0.03 MB DOC)

Appendix S7 PBS values of individual gene.
(0.02 MB XLS)
