# Supplementary material for: Phylogenomic Analysis Resolves the Formerly Intractable Adaptive Diversification of the Endemic Clade of East Asian Cyprinidae (Cypriniformes)
Source: PLoS One. 2010 Oct 20;5(10):e13508. doi: 10.1371/journal.pone.0013508 (PMC2958143; doi:10.1371/journal.pone.0013508)
Supplement: Appendix S1 — Primers for PCR amplification of the sampled genes in present study. (0.12 MB DOC) [file pone.0013508.s001.doc]

Additional data file 1

Primers for PCR amplification of the sampled genes in present study

| Gene name | Primer F | Primer R |
| --- | --- | --- |
| Ptr (si:ch211-105n9.1), hypothetical protein LOC564097 | 1st: AGAATGGATWACCAACACYTACG ^1^  2nd: GGATAACCAACACYTACGTCAA ^1^ | 1st: TAAGGCACAGGATTGAGATGCT ^1^  2nd: ACAGGATTGAGATGCTGTCCA ^1^ |
| T-box 1, brain (tbr1) | 1st:TGTCTACACAGGCTGCGACAT ^1^  2nd:GCCATGMCTGGYTCTTTCCT ^1^ | 1st:GATGTCCTTRGWGCAGTTTTT ^1^  2nd:GGAGCAGTTTTTCTCRCATTC ^1^ |
| myosin, heavy polypeptide 6 | 1st :CATMTTYTCCATCTCAGATAATGC ^1^  2nd:GGAGAATCARTCKGTGCTCATCA ^1^ | 1st:ATTCTCACCACCATCCAGTTGAA ^1^  2nd:CTCACCACCATCCAGTTGAACAT ^1^ |
| ectodermal-neural cortex 1-like protein (ENC1) gene | 1st:GACATGCTGGAGTTTCAGGA ^1^  2nd:ATGCTGGAGTTTCAGGACAT ^1^ | 1st:ACTTGTTRGCMACTGGGTCAAA ^1^  2nd:AGCMACTGGGTCAAACTGCTC ^1^ |
| sorting nexin 33 | 1st: GTATGGTSGGCAGGAACYTGAA ^1^  2nd: GACGTTCCCATGATGGCWAAAAT ^1^ | 1st: CAAACAKCTCYCCGATGTTCTC ^1^  2nd: CATCTCYCCGATGTTCTCGTA ^1^ |
| glycosyltransferase | 1st :GGACTGTCMAAGATGACCACMT ^1^  2nd: ACATGGTACCAGTATGGCTTTGT ^1^ | 1st: CCCAAGAGGTTCTTGTTRAAGAT ^1^  2nd: GTAAGGCATATASGTGTTCTCTCC ^1^ |
| pleiomorphic adenoma gene-like 2 | 1st: CCACACACTCYCCACAGAA ^1^  2nd: AAAAGATGTTTCACCGMAAAGA ^1^ | 1st: TTCTCAAGCAGGTATGAGGTAGA ^1^  2nd: GGTATGAGGTAGATCCSAGCTG ^1^ |
| Super conserved receptor expressed in brain 2. | 1st: ATGGCGAACTAYAGCCATGC ^1^  2nd: TGCAGGGGACCACAMCAT ^1^ | 1st :CTGGATTTTCTGCAGTASAGGAG ^1^  2nd: CAGTASAGGAGCGTGGTGCT ^1^ |
| zic family member 1 | 1st: GGACGCAGGACCGCARTAYC ^1^  2nd: GGACCGCAGTATCCCACYMT1 | 1st: CTGTGTGTGTCCTTTTGTGRATYTT ^1^  2nd: GTGTGTCCTTTTGTGAATTTTYAGRT1 |
| similar to SH3 and PX domain  containing 3 gene | 1st: GTATGGTSGGCAGGAACYTGAA ^1^  2nd: GACGTTCCCATGATGGCWAAAAT ^1^ | 1st: CAAACAKCTCYCCGATGTTCTC ^1^  2nd: CATCTCYCCGATGTTCTCGTA ^1^ |
| HoxA1a | ATGAGCASMTTCTTAGAYTWTTCG^2^ | GACTTYTCAGAYGCATCMTCKGTT^2^ |
| HoxA2a | ATGAATTACGAATTYGRGCGAGA^2^ | CCTTKMGGRGAGAAGCAYASAGG^2^ |
| HoxA2b | ATGAATTACGAATTYGRGCGAGA^2^ | GTKTTGCWGGTCSATTGYTGT^2^ |
| HoxA4a | GAGATCAGAAWAACGACAGM^2^ | ACCAGATCTTCACCTGACGYTC^2^ |
| HoxA5a | GGCTACAATGGMATGGATCTCAG^2^ | TCCTGCMGCKGCCATRYTCATRCTT^2^ |
| HoxA9a | TGCCATGTCGACATCCGGA^2^ | TTCMSCTTCATCCKGCGGT^2^ |
| HoxB1a | GGAGTACACAATTTGYAACCG^2^ | TTAGGAATCSGGACTTGGAGA^2^ |
| HoxB3a | GCAGCRGTGGDGAGAARAGYCC^2^ | CTTGGGTGCTTCYTGGATTCT^2^ |
| HoxB5a | CGACCAMGATCYACAAATCAAG^2^ | ATGGCTAATATGTAGTTTCCTC^2^ |
| HoxB6b | GGAGCWACCAAYGTSCARGA^2^ | AGRGTCTGRAASCGRGTGTA^2^ |
| HoxB8a | ATGAGCTCWTATTTCGTCAACTC^2^ | TTGCTGCTKGGRAACTTGTC^2^ |
| HoxB8b | ATGAGTTCCTACTTCGTCAATTC^2^ | TTGCTGCTKGGRAACTTGTC^2^ |
| HoxB9a-F | ATGTCCATTTCTGGRACYCT^2^ | TAKTCYTTGGAYTGGTCCTT^2^ |
| HoxB13a | TGGTCCTGAATCCTCGCT^2^ | CTAHGGCGCGVTGYTTTTMAC^2^ |
| HoxC1a | GAATTCTTATCATGGGTTCAGGG^2^ | CACGGGACTGATTTCTCCT^2^ |
| HoxC8a | ATGAGCTCCTAYTTYGTYAAYCC^2^ | GGCTGTAWGTYTGCCKTCCAYT^2^ |
| HoxC9a | ATGTCGDCYACGGGTCCCATAA^2^ | GGTCTAATTCHGSCTTSTCGTC^2^ |
| HoxC12b | ATGGGCGAGCATAATCTCYTT^2^ | GTARGMBAAAGCYTGCTCYCKC^2^ |
| HoxC12a | ATGGGCGAGCATAATCTCYTT^2^ | CATYCGMCGATTTTGGAACCA^2^ |
| HoxC13a | ATGACGACTTCGCWRSTYCTG^2^ | GGDGATTTCCADAGRTGAG^2^ |
| HoxD3a-F | ATGCAGAAAGCMACHTATTAYGAC^2^ | TTACAGRTGCGTSAGTTTRGG ^2^ |
| HoxD4a-F | TGACAGCAAGTAGGAGGGCTTTATG^2^ | CGCCGCCTKGTCAGATACCTGTT^2^ |
| HoxD9a | ATGASTWCGAAGGGATYCAAAMATG^2^ | CTGTTCTGRAACCAGATYTTSAC^2^ |
| HoxD10a | ATGTCCTTYCCCARCAGCTCTC^2^ | CATCCTGCGGTTYTGRAACCAG^2^ |
| HoxD10b | ATGTCCTTYCCCARCAGCTCTC^2^ | TCGCRTAWGTTTGGCTCAGTC^2^ |
| HoxD11a | ATGACGGAMTACGATGATCGCA^2^ | CAGAACAAWGGRTTYCCAGTG^2^ |
| HoxD12a | CCGTGGTGYYCVTCRCARGTGA^2^ | GAARGYHTGCTCBCGCATCATSAG^2^ |
| 28sd2 | GGTGGTAAACTCCATCTAAGGCTA^3^ | ATAGTTCACCATCTTTCGGGTCC^3^ |
| beta-actin gene | CGACATGGAGAAGATCTGGCA | GGCTGTGATCTCCTTCTGCAT |
| Recombination activating gene 1 | CTGAGCTGCAGTCAGTACCATAAGATGT^4^ | TGAGCCTCCATGAACTTCTGAAGRTAYTT^4^ |
| Growth hormone | YTGTCKDTGGTSCTGGTYAG^7^ | GCTYTTYTGBGTTTCATSTTT^7^ |
| rhodopsin | TACGTGCCTATGTCCAAYGC^5^ | TGCTTGTTCATGCAGATGTAGA^6^ |
| interphotoreceptor retinoid-binding protein gene | AACTACTGCTCRCCAGAAAARC^5^ | GGAAATGCATAGTTGTCTGCAA^5^ |
| Early growth response protein gene 1 | TMTCTTACACAGGCCGYTTCAC^5^ | CTTTYTCTGCTTTCTTGTCCTTCT^5^ |
| Early growth response protein gene 2b | AGTTTTCCATCGACTCSCAGTA^5^ | AGGTGGATTTTGGTGTGTCTYTT^5^ |
| Early growth response protein gene 3 | AATATCATGGACYTGGGNATGG^5^ | GGYTTCTTGTCCTTCTGTTTSAG^5^ |
| apolipoprotein | GGTWSCCAGGCNBDKYYYMTGCAGG | GGCAWVAGCTTGGHCTTGGTCTCCTC |
| si:ch211-105n9.1 hypothetical protein | GCCAGCCCACTTGGTTTA | GTCTACATTCCAGAGCGTCA |
| zinc finger and BTB domain containing 22 | CCTCGGAAGACTACCTC | CCCACTACGATTGCCACCGATGCCC |
| adenylosuccinate lyase part1 | TCTTCTGGCTGGGATACTTC | GCCATAGCAGTCTGTGAGG |
| adenylosuccinate lyase part2 | AGATTGAGGAGCCGTTTG | CACCTTAGGATAGACCACC |
| c-Jun | CTSATMATCCAGTCYAGCAAC | CTGTTBACGTGGTTCATGAC |
| caudal type homeo box transcription factor 4 | TTAATCGCTACATCACAA | TTTAGATACACTCACCACAT |
| Gastrulation brain homeobox 1 | TTTCACTAGCGAACAGCT | TAGACAACAAATGGCAAC |
| luteinizing hormone | TTCTGTCTGTATTCGGTGGT | CTGCGGTCGAGGTGTTAG |
| kelch-like 11 | CACATGTAYACYYTGRRTCAG | CCATGRCCNCCRATRCTATASAG |
| New Hypothetical protein(rh2) | GCTGAACCTCTGGCTCTA | ACGAATCGGTAATGTCTT |
| inhibin, beta B | AGGCAAATCTGTGGCTGTA | GGTGCTGAGTTTAGTGGGTAT |
| sphingosine 1-phosphate receptor (edg1) | TACTTCATTGGGAACTTGG | GTCAGGGTGTAGATCAGAGG |
| somatostatin receptor | TCCAACCTGTCCCTCCCTGAC | CCATTGAGCGCGGCGTT |
| dermatan 4 sulfotransferase 1 | ACTGCTACGTGCCCAAAG | AACAGGGAGAAGTCCAAA |
| alpha-1-microglobulin/bikunin | CATGTAAGGCTGAGCCAGATTC | CGGGTNTCYATTTGTG |
| opsin 1 | TGGGAAAAGATGAACGGC | CATGAAGCCTTCAATCGC |
| gonadotropin alpha subunit | AGATATGCTGGAGCAAGTA | GAACAATCTTATAGCAGCGT |
| kelch repeat and BTB (POZ) domain | TGGATGATAACGTGCAGGAG | GAGCTGGTTCTTCTTGGTGC |
| V1r pheromone receptor-like | AAAGGCGTCTCCTTCCTGCTGCAGGCTGGTCTG | CGCTTTCACCTTCCYGTTGGAGGAGATGAT |
| calcium sensing receptor part2 | TAAATGCCCAAACAACTCC | TCAACAGCCGAGACGAAT |
| zinc finger and BTB domain | GTGCTGCTGAAGAACAT | TCCTGACACCACGATC |
| connexin 52.6 | CTTGCTTGGGAGTATCTTAG | AATGCTGCTGGTATTTCTAT |
| one-eyed pinhead | CAGGGATTGTGGTGTTAT | ATGGACGACTTGTTTGTAT |
| Hypothetical protein | GGTWSCCAGGCNBDKYYYMTGCAGG | GGCAWVAGCTTGGHCTTGGTCTCCTC |
| periplakin | TGCGTGGGATTGAGACCG | TTCCGCTTGAGGCTTTCG |
| calcium sensing receptor part1 | GACAATCCCTACAGACGAG | GCATAAACAGCCACATACA |
| tripartite motif-containing 9 | CATCGGCGTCACAGGAAA | GAGCGGCGGAAGTTGTCT |
| melanocortin 4 receptor | AAGCTTATGAACACCTCACATCAC | GATCTCCTTGAAGGTCTTCC |
| interphotoreceptor (IRBP1) | ATAYCTGCGYTTKGACAGGTT | GACTGBATYARGTAAGCAAACTC |
| alpha-1D adrenoreceptor | GGGGTAAAACACTGCCTGAA | GGGCAGAAGTCTCTGTGAGG |
| amylase, alpha 2A | CGGAAATGAAAATGAGCT | CTTGGCACCATACTTGAA |
| wingless-type MMTV integration site family | ACGGGATCCTACAAGTGC | GGAAAGCYGAGATTCTTGG |
| dermacan | CACCGAACAGGAGGCTAGAG | CGTCGGTAGCCACCATATCT |
| alpha-1D adrenoreceptor3 | GGGGTAAAACACTGCCTGAA | GGGCAGAAGTCTCTGTGAGG |
| mediator complex subunit 7 | GAGCCACAGCAGGTCAGTGC | GTGGCAAATCATCAGGCAG |
| growth hormone | TGYTTYAARAARGAYATG | AGRTANGTYTCNACCT |
| Tbx21 tbet | GACCGCCTYACRCCWTCCC | TCAGTGGGTGTAGAAGCC |
| myogenic differentiation 1 | TGTCGGATATYCCBTTCCCC | AGTAVGWGCTGTCATAGCTG |
| 28S ribosomal RNA gene | AGCGGAGGAAAAGAAACTA | TACTAGAAgGTTCGATTAGTC |
| alpha-tubulin | GGTGCTGGTAAYAAYTGRGC | ACCAGGTCGTTCATRTTNGA |

AIUPAC codes: R = A/G; Y = C/T; K=G/T; S = G/C; W = A/T; M = A/C; B = C/G/T; D = A/G/T; H = A/C/T and V = A/C/G; N=A/C/G/T.

Note: Primers of two genes manganese superoxide dismutase and eukaryotic translation initiation factor 1B (eif1b) were available upon request.

**References**

1. Li C, Orti G, Zhang G, Lu G (2007) A practical approach to phylogenomics: the phylogeny of ray-finned fish (Actinopterygii) as a case study. BMC Evol Biol 7:44.

2. Zou SM, Jiang XY, He ZZ, Yuan J, Yuan XN, Li SF (2007) Hox gene clusters in blunt snout bream, Megalobrama amblycephala and comparison with those of zebrafish, fugu and medaka genomes. Gene 400:60-70.

3. Zardoya R, Meyer A (1996) Evolutionary relationships of the coelacanth, lungfishes, and tetrapods based on the 28S ribosomal RNA gene. Proc Natl Acad Sci U S A 93:5449-5454.

4. J. Andrés López W-JC, Guillermo Ortí (2004) Esociform Phylogeny. Copeia 2004(3):449-464.

5. Chen WJ, Miya M, Saitoh K, Mayden RL (2008) Phylogenetic utility of two existing and four novel nuclear gene loci in reconstructing Tree of Life of ray-finned fishes: the order Cypriniformes (Ostariophysi) as a case study. Gene 423:125-134.

6. Chen WJ, Bonillo C, Lecointre G (2003) Repeatability of clades as a criterion of reliability: a case study for molecular phylogeny of Acanthomorpha (Teleostei) with larger number of taxa. Mol Phylogenet Evol 26:262-288.

7. Clements MD, Bart HL, Jr., Hurley DL (2004) Isolation and characterization of two distinct growth hormone cDNAs from the tetraploid smallmouth buffalofish (Ictiobus bubalus). General and comparative endocrinology 136:411-418.
