# Supplementary material for: Phylogenomic Analysis Resolves the Formerly Intractable Adaptive Diversification of the Endemic Clade of East Asian Cyprinidae (Cypriniformes)
Source: PLoS One. 2010 Oct 20;5(10):e13508. doi: 10.1371/journal.pone.0013508 (PMC2958143; doi:10.1371/journal.pone.0013508)
Supplement: Appendix S2 — A list of Genbank accession numbers of the sequences of nuclear genes sampled in this study. (0.28 MB DOC) [file pone.0013508.s002.doc]

Additional data file 2

A list of Genbank accession numbers of the sequences of nuclear genes sampled in this study. A- *Mylopharyngodon piceus*; B- *Ctenopharyngodon idella*; C- *Hypophthalmichthys molitrix*; D- *Hypophthalmichthys nobilis*; E- *Squaliobarbus curriculus*；F- *Megalobrama amblycephala*； G- *Elopichthys bambusa*; H- *Xenocypris argentea*； I- *Culter alburnus*； J- *Opsariichthys bidens*；K- *Ochetobius elongatus*; L- *Luciobrama macrocephalus*.

| Gene order^ψ^ | Genbank accession numbers | | | | | | | | | | | |
| --- | --- | --- | --- | --- | --- | --- | --- | --- | --- | --- | --- | --- |
|  | A | B | C | D | E | F | G | H | I | J | K | L |
| Ptr (si:ch211-105n9.1) hypothetical protein LOC564097 | GU218551 | GU218552 | GU218553 | GU218554 | GU218555 | GU218556 | GU218557 | GU218558 | GU218559 | GU218560 | GU218561 | GU218562 |
| T-box 1, brain (tbr1) | GU218563 | GU218564 | GU218565 | GU218566 | GU218567 | GU218568 | GU218569 | GU218570 | GU218571 | GU218572 | GU218573 | GU218574 |
| early growth response 2B | GU218575 | GU218576 | GU218577 | GU218578 | GU218579 | GU218580 | GU218581 | GU218582 | GU218583 | GU218584 | GU218585 | GU218586 |
| rhodopsin | GU218587 | GU218588 | GU218589 | GU218590 | GU218591 | GU218592 | GU218593 | GU218594 | GU218595 | GU218596 | GU218597 | GU218598 |
| large subunit ribosomal RNA gene | GU218599 | --------- | ---------- | ---------- | ---------- | ---------- | GU218600 | GU218601 | GU218602 | ---------- | GU218603 | GU218604 |
| beta-actin gene | GU218605 | GU218606 | GU218607 | ---------- | GU218608 | GU218609 | ---------- | ---------- | ---------- | ---------- | ---------- | ---------- |
| Myosin heavy polypeptide 6 | GU218610 | GU218611 | GU218612 | GU218613 | GU218614 | GU218615 | GU218616 | GU218617 | GU218618 | GU218619 | GU218620 | GU218621 |
| recombination activating gene 2 | DQ367011 | DQ366996 | DQ367002 | DQ367038 | DQ367021 | DQ367025 | DQ367016 | DQ367024 | DQ367004 | DQ367014 | DQ367012 | DQ367013 |
| sorting nexin 33 | GU218622 | GU218623 | GU218624 | GU218625 | GU218626 | GU218627 | GU218628 | GU218629 | GU218630 | GU218631 | GU218632 | GU218633 |
| ectodermal-neural cortex 1-like protein (ENC1) gene | GU217798 | GU217799 | GU217800 | GU217801 | GU217802 | GU217803 | GU217804 | GU217805 | GU217806 | GU217807 | GU217808 | GU217809 |
| glycosyltransferase | GU218634 | GU218635 | GU218636 | GU218637 | GU218638 | GU218639 | GU218640 | GU218641 | GU218642 | GU218643 | GU218644 | GU218645 |
| beta-actin gene2 | GU217810 | GU217811 | GU217812 |  | GU217813 | GU217814 | GU217815 | GU217816 | GU217817 |  | GU217818 | GU217819 |
| homeodomain protein c1a | GU217820 | GU217821 | GU217822 | GU217823 | GU217824 | GU217825 | EF197773 | GU217826 | GU217827 | GU217828 | GU217829 | GU217830 |
| Recombination activating gene 1 | GU217831 | GU217832 | GU217833 | GU217834 | GU217835 | GU217835 | GU217836 | GU217837 | GU217838 | GU217839 | GU217840 | GU217841 |
| patched domain-containing protein 1, partial cds | GU217843 | GU217844 | GU217845 | GU217846 | GU217847 | GU217848 | GU217849 | GU217850 | GU217851 | GU217852 | GU217853 | GU217854 |
| zinc finger and BTB domain containing 22,transcript variant | GU218680 | GU218681 | GU218682 | GU218683 | GU218684 | GU218685 | GU218686 | GU218687 | GU218688 | GU218689 | GU218690 | GU218691 |
| homeodomain protein  A9a | GU217855 | GU217856 | GU217857 | GU217858 | GU217859 | EF197748 | GU217860 | GU217861 | GU217862 | GU217863 | GU217864 | GU217865 |
| S7 ribosomal protein gene, intron 1 | DQ163926 | AY325787 | AY325778 | AY325774 | AY103170 | AY325779 | AY325775 | DQ163930 | AY103165 | ---------- | AY325776 | AY325780 |
| homeodomain protein  A2b | GU217866 | GU217867 | GU217868 | ---------- | GU217869 | EF197744 | GU217870 | GU217871 | GU217872 | GU217873 | ---------- | GU217874 |
| homeodomain protein  D9a | GU217875 | GU217876 | GU217877 | GU217878 | GU217879 | EF197786 | GU217880 | GU217881 | GU217882 | GU217883 | GU217884 | GU217885 |
| adenylosuccinate lyase part1 | ---------- | GU217886 | GU217887 | GU217888 | GU217889 | ---------- | GU217890 | ---------- | ---------- | GU217891 | ---------- | ---------- |
| c-Jun | GU217892 | GU217893 | GU217894 | GU217895 | GU217896 | GU217897 | GU217898 | GU217899 | GU217900 | GU217901 | GU217902 | ---------- |
| caudal type homeo box transcription factor 4 | GU217903 | GU217904 | GU217905 | GU217906 | GU217907 | GU217908 | GU217909 | GU217910 | GU217911 | GU217912 | GU217913 | GU217914 |
| adenylosuccinate lyase part1 | GU217915 | GU217916 | GU217917 | GU217918 | GU217919 | GU217920 | GU217921 | GU217922 | GU217923 | GU217924 | GU217925 | GU217926 |
| Gastrulation brain homeobox 1 (gbx1) | GU217927 | GU217928 | GU217929 | GU217930 | GU217931 | GU217932 | GU217933 | GU217934 | GU217935 | GU217936 | GU217937 | GU217938 |
| luteinizing hormone | GU217939 | GU217940 | GU217941 | GU217942 | ---------- | GU217943 | GU217944 | GU217945 | GU217946 | GU217947 | ---------- | GU217948 |
| kelch-like 11 | GU217949 | GU217950 | GU217951 | GU217952 | GU217953 | GU217954 | GU217955 | GU217956 | ---------- | ---------- | ---------- | ---------- |
| clone PMB18-T RH | GU217957 | ---------- | ---------- | GU217958 | GU217959 | ---------- | ---------- | ---------- | ---------- | ---------- | GU217960 | ---------- |
| homeodomain protein A13a | ---------- | ---------- | GU217961 | GU217961 | GU217961 | EF197753 | ---------- | GU217961 | GU217961 | GU217961 | ---------- | ---------- |
| homeodomain protein B5b | GU217967 | GU217968 | GU217969 | GU217970 | EF197761 | GU217971 | GU217972 | GU217973 | GU217974 | GU217975 | GU217976 | GU217977 |
| inhibin, beta B | GU217978 | GU217979 | GU217980 | GU217981 | GU217982 | GU217983 | GU217984 | GU217985 | GU217986 | GU217987 | GU217988 | GU217989 |
| sphingosine 1-phosphate receptor (edg1) | GU217990 | GU217991 | GU217992 | GU217993 | GU217994 | GU217995 | GU217996 | GU217997 | ---------- | GU217998 | GU217999 | GU21800 |
| homeodomain protein D4a | GU218001 | GU218002 | GU218003 | GU218004 | GU218005 | EF197785 | GU218006 | GU218007 | GU218008 | GU218009 | GU218010 | GU218011 |
| somatostatin receptor 2 | GU218012 | GU218013 | GU218014 | GU218015 | GU218016 | ---------- | GU218017 | ---------- | GU218018 | ---------- | GU218019 | ---------- |
| dermatan 4 sulfotransferase 1 | GU218020 | GU218021 | ---------- | ---------- | GU218022 | GU218023 | GU218024 | GU218025 | ---------- | GU218026 | GU218027 | ---------- |
| homeodomain protein (HoxB6b) | GU218028 | GU218029 | GU218030 | GU218031 | GU218032 | EF197763 | GU218033 | GU218034 | GU218035 | GU218036 | GU218037 | GU218038 |
| alpha-1-microglobulin | GU218039 | GU218040 | GU218041 | ---------- | ---------- | GU218042 | ---------- | ---------- | ---------- | ---------- | GU218043 | ---------- |
| opsin 1 | GU218044 | GU218045 | GU218046 | GU218047 | GU218048 | GU218049 | GU218050 | GU218051 | GU218052 | ---------- | GU218053 | GU218054 |
| homeo box B1b | ---------- | ---------- | GU218055 | GU218056 | ---------- | EF197756 | GU218057 | ---------- | GU218058 | ---------- | ---------- | ---------- |
| gonadotropin alpha subunit | GU218059 | ---------- | GU218060 | ---------- | ---------- | GU218061 | GU218062 | GU218063 | ---------- | ---------- | GU218064 | GU218065 |
| homeo box B1a | GU218066 | GU218067 | GU218068 | GU218069 | GU218070 | EF197755 | GU218071 | GU218072 | GU218073 | GU218074 | GU218075 | GU218076 |
| homeo box B3a | GU218077 | GU218078 | GU218079 | GU218080 | GU218081 | EF197758 | GU218082 | GU218083 | GU218084 | GU218085 | GU218086 | GU218087 |
| kelch repeat and BTB (POZ) domain containing 10 (kbtbd10) | GU218088 | GU218089 | GU218090 | GU218091 | GU218092 | GU218093 | GU218094 | GU218095 | GU218096 | GU218097 | GU218098 | GU218099 |
| V1r pheromone receptor-like (V1r2) gene | GU218100 | GU218101 | GU218102 | GU218103 | GU218104 | GU218105 | GU218106 | GU218107 | GU218108 | GU218109 | GU218110 | GU218111 |
| DNA sequence from clone casf2 | ---------- | GU218112 | GU218113 | GU218114 | ---------- | GU218115 | ---------- | GU218116 | GU218117 | ---------- | ---------- | ---------- |
| zinc finger and BTB domain containing 22, transcript variant 1 | ---------- | GU218673 | GU218674 | GU218675 | ---------- | ---------- | GU218676 | ---------- | ---------- | GU218677 | GU218678 | GU218679 |
| connexin 52.6 gene | GU218118 | GU218119 | GU218120 | GU218121 | GU218122 | GU218123 | ---------- | GU218124 | GU218125 | ---------- | ---------- | ---------- |
| homeodomain protein (HoxC12b) gene | GU218126 | GU218127 | GU218128 | GU218129 | ---------- | EF197781 | ---------- | ---------- | GU218130 | ---------- | ---------- | ---------- |
| homeodomain protein (HoxC12a) | GU218131 | GU218132 | ---------- | GU218133 | GU218134 | ---------- | GU218135 | GU218136 | GU218137 | GU218138 | ---------- | ---------- |
| interphotoreceptor retinoid-binding protein | GU218661 | GU218662 | GU218663 | GU218664 | GU218665 | GU218666 | GU218667 | GU218668 | GU218669 | GU218670 | GU218671 | GU218672 |
| early growth response 3 (EGR3) | GU218139 | GU218140 | GU218141 | GU218142 | GU218143 | GU218144 | GU218145 | GU218146 | GU218147 | GU218148 | GU218149 | GU218150 |
| early growth response 2B (EGR2B) gene | GU218151 | GU218152 | GU218153 | GU218154 | GU218155 | GU218156 | GU218157 | GU218158 | GU218159 | GU218160 | GU218161 | GU218162 |
| one-eyed pinhead, intron | GU218163 | GU218164 | GU218165 | GU218166 | GU218167 | GU218168 | GU218169 | GU218170 | GU218171 | GU218172 | GU218173 | GU218174 |
| apolipoprotein A-I | GU218175 | ---------- | GU218176 | GU218177 | GU218178 | GU218179 | GU218180 | GU218181 | GU218182 | ---------- | GU218183 | ---------- |
| apolipoprotein A-I protein(apoA1 gene) | ---------- | ---------- | ---------- | GU218184 | GU218185 | GU218186 | GU218187 | GU218188 | GU218189 | GU218190 | GU218191 | GU218192 |
| periplakin | GU218193 | GU218194 | GU218195 | GU218196 | GU218197 | GU218198 | GU218199 | GU218200 | GU218201 | ---------- | GU218202 | GU218203 |
| homeodomain protein (HoxA1a) | GU218204 | GU218205 | GU218206 | GU218207 | GU218208 | EF197742 | ---------- | ---------- | GU218209 | GU218210 | GU218211 | GU218212 |
| homeodomain protein (HoxC4a) | GU218213 | GU218214 | GU218215 | GU218216 | GU218217 | EF197771 | ---------- | ---------- | GU218218 | GU218219 | GU218220 | GU218221 |
| homeodomain protein (HoxC8a) | GU218222 | GU218223 | GU218224 | GU218225 | GU218226 | EF197775 | ---------- | ---------- | GU218227 | GU218228 | GU218229 | GU218230 |
| similar to extracellular calcium-sensing receptor | ---------- | ---------- | ---------- | GU218231 | ---------- | ---------- | ---------- | ---------- | ---------- | GU218232 | GU218233 | GU218234 |
| DNA sequence from clone trim9 | GU218235 | GU218236 | GU218237 | GU218238 | ---------- | ---------- | ---------- | ---------- | ---------- | ---------- | GU218239 | GU218240 |
| homeodomain protein (HoxA5a) | GU218241 | GU218242 | GU218243 | GU218244 | GU218245 | EF197747 | GU218246 | GU218247 | ---------- | GU218248 | GU218249 | ---------- |
| homeo box C13a (hoxc13a) | ---------- | GU218250 | GU218251 | GU218252 | GU218253 | EF197782 | GU218254 | GU218255 | GU218256 | GU218257 | GU218258 | GU218259 |
| melanocortin 4 receptor | GU218260 | GU218261 | GU218262 | GU218263 | GU218264 | GU218265 | GU218266 | GU218267 | GU218268 | GU218269 | GU218270 | GU218271 |
| interphotoreceptor retinoid-binding protein | GU218649 | GU218650 | GU218651 | GU218652 | GU218653 | GU218654 | GU218655 | GU218656 | GU218657 | GU218658 | GU218659 | GU218660 |
|  |  |  |  |  |  |  |  |  |  |  |  |  |
| similar to alpha-1D adrenoreceptor | GU218272 | GU218273 | GU218274 | GU218275 | ---------- | GU218276 | ---------- | GU218277 | GU218278 | GU218289 | GU218280 | GU218281 |
| amylase | GU218282 | GU218283 | GU218284 | GU218285 | GU218286 | GU218287 | ---------- | GU218288 | GU218289 | GU218290 | ---------- | GU218291 |
| ingless-type MMTV integration site family, member 8b (wnt8b) | GU218292 | GU218293 |  | GU218294 | GU218295 | GU218296 | GU218297 | GU218298 | GU218299 | GU218300 | GU218301 | GU218302 |
| dermacan | GU218303 | GU218304 | GU218305 | GU218306 | GU218307 | GU218308 | GU218309 | GU218310 | GU218311 | GU218312 | ---------- | GU218313 |
| glycoprotein hormones, alpha polypeptide | GU218314 | GU218315 | GU218316 | GU218317 | ---------- | GU218318 | GU218319 | GU218320 | GU218321 | ---------- | ---------- | ---------- |
| similar to alpha-1D adrenoreceptor | GU218322 | GU218323 | GU218324 | GU218325 | ---------- | GU218326 | GU218327 | GU218328 | GU218329 | GU218330 | GU218331 | GU218332 |
| mediator complex subunit 7 | GU218333 | GU218334 | GU218335 | GU218336 | GU218337 | GU218338 | GU218339 | GU218340 | GU218341 | GU218342 | GU218343 | GU218344 |
| growth hormone (GH) | GU218345 | GU218346 | GU218347 | GU218348 | GU218349 | ---------- | ---------- | GU218350 | GU218351 | GU218352 | GU218353 | GU218354 |
| T-box 21 | GU218355 | GU218356 | GU218357 | GU218358 | GU218359 | GU218340 | GU218341 | GU218342 | GU218343 | GU218344 | GU218345 | GU218346 |
| homeodomain protein (HoxB9a) gene | GU218365 | ---------- | ---------- | GU218366 | GU218367 | EF197767 | GU218368 | ---------- | GU218369 | GU218370 | GU218371 | GU218372 |
| homeo box D3a (hoxd3a) | GU218373 | GU218374 | GU218375 | GU218376 | ---------- | EF197784 | ---------- | ---------- | GU218377 | GU218378 | GU218379 | GU218380 |
| homeo box D12a (hoxd12a) | GU218381 | GU218382 | GU218383 | ---------- | GU218384 | EF197789 | ---------- | ---------- | GU218385 | GU218386 | GU218387 | GU218388 |
| homeodomain protein (HoxC9a) | ---------- | GU218389 | GU218390 | GU218391 | GU218392 | EF197776 | GU218394 | GU218395 | GU218396 | GU218397 | GU218398 | GU218399 |
| Cellular myelocytomatosis oncogene | EF194851 | EF194850 | EF194849 | EF194848 | EF194854 | EF194856 | EF194853 | EF194859 | EF194855 | EF194861 | EF194852 | ---------- |
| homeodomain protein (HoxB8a) gene | GU218400 | GU218401 | GU218402 | GU218403 | GU218404 | EF197765 | GU218405 | GU218406 | GU218407 | GU218408 | ---------- | GU218409 |
| homeo box C10a (hoxc10a) | GU218410 | GU218411 | GU218412 | GU218413 | GU218414 | EF197777 | GU218415 | GU218416 | GU218417 | GU218418 | GU218419 | GU218420 |
| homeodomain protein (HoxB13a) gene | GU218421 | GU218422 | GU218423 | GU218424 | GU218425 | EF197768 | GU218426 | GU218427 | ---------- | GU218428 | ---------- | GU218429 |
| similar to homeobox protein HoxC13b | GU218430 | GU218431 | GU218432 | GU218433 | GU218434 | EF197783 | GU218435 | GU218436 | GU218437 | GU218438 | ---------- | GU218439 |
| homeodomain protein B5a | GU218440 | GU218441 | GU218442 | GU218443 | GU218444 | EF197760 | GU218445 | GU218446 | GU218447 | GU218448 | GU218449 | GU218450 |
| homeodomain protein (HoxB8b) | GU218451 | GU218452 | GU218453 | GU218454 | GU218455 | EF197766 | GU218456 | GU218457 | GU218458 | ---------- | GU218459 | GU218460 |
| myogenic differentiation 1 | GU218461 | GU218462 | GU218463 | GU218464 | GU218465 | GU218466 | GU218467 | GU218468 | GU218469 | GU218470 | GU218471 | GU218472 |
| 28S ribosomal RNA gene | ---------- | ---------- | ---------- | ---------- | ---------- | GU218546 | GU218547 | GU218548 | GU218549 | GU218550 | ---------- | ---------- |
| ryanodine receptor 3 | GU218473 | GU218474 | GU218475 | GU218476 | GU218477 | GU218478 | GU218479 | GU218480 | GU218481 | GU218482 | GU218483 | GU218484 |
| homeodomain protein (HoxD11a) | GU218485 | GU218486 | GU218487 | GU218488 | GU218489 | EF197788 | GU218490 | GU218491 | GU218492 | GU218493 | GU218494 | GU218495 |
| homeodomain protein (HoxD10a) | GU218496 | GU218497 | GU218498 | GU218499 | GU218500 | EF197787 | GU218501 | GU218502 | GU218503 | GU218504 | GU218505 | GU218506 |
| ribosomal protein S6 kinase 1 (S6K1) gene | ---------- | EF373673 | EF373672 | EF373671 | ---------- | EF373669 | EF373674 | EF373670 | ---------- | EF373675 | ---------- | ---------- |
| DNA sequence from clone mapk | GU218646 | GU218647 | GU218648 | ---------- | ---------- | ---------- | ---------- | ---------- | ---------- | ---------- | ---------- | ---------- |
| SREB2 | ---------- | GU218507 | GU218508 | GU218509 | GU218510 | GU218511 | GU218512 | GU218513 | GU218514 | GU218515 | GU218516 | GU218517 |
| connective tissue growth factor (CTGF) gene | ---------- | ---------- | EF524099 | EF524098 | ---------- | EF524097 | EF524102 | ---------- | ---------- | EF524103 | EF524101 | ---------- |
| alpha-tubulin | ---------- | GU218518 | ---------- | GU218519 | ---------- | GU218520 | GU218521 | GU218522 | GU218523 | ---------- | ---------- | ---------- |
| pleiomorphic adenoma gene-like 2 | GU218524 | GU218525 | GU218526 | GU218527 |  |  | GU218528 | GU218529 | GU218530 | GU218531 | GU218532 | GU218533 |
| insulin receptor a | ---------- | EU009542 | ---------- |  | EU009541 | EU009544 | ---------- | ---------- | EU009543 | EU009540 | ---------- | ---------- |
| insulin receptor b | EU009598 | EU009583 | ---------- | EU009592 | EU009584 | EU009589 | EU009586 | EU009590 | EU009588 | EU009578 | EU009585 | ---------- |
| manganese superoxide dismutase | ---------- | GU218534 | ---------- | GU218535 | GU218536 | ---------- | ---------- | ---------- | GU218537 | ---------- | GU218538 | ---------- |
| eukaryotic translation initiation factor 1B (eif1b) | ---------- | GU218539 | GU218540 | GU218541 | GU218542 | ---------- | ---------- | GU218543 | GU218544 | ---------- | GU218545 | ---------- |

---------- not available
