# Supplementary material for: Phylogenomic Analysis Resolves the Formerly Intractable Adaptive Diversification of the Endemic Clade of East Asian Cyprinidae (Cypriniformes)
Source: PLoS One. 2010 Oct 20;5(10):e13508. doi: 10.1371/journal.pone.0013508 (PMC2958143; doi:10.1371/journal.pone.0013508)
Supplement: Appendix S3 — Ensembl gene accession numbers of predicted single-copy nuclear genes extracted from genome sequences of Danio rerio. (0.06 MB DOC) [file pone.0013508.s003.doc]

Additional file 3

Ensembl gene accession numbers of predicted single-copy nuclear genes extracted from genome sequences of *Danio rerio*.

Single-copy genes with lengths of exon > 800bp

ENSDARP00000088854

ENSDARP00000023810

ENSDARP00000019822

ENSDARP00000088099

ENSDARP00000059142

ENSDARP00000088230 luteinizing hormone/choriogonadotropin receptor

ENSDARP00000036643 luteinizing hormone/choriogonadotropin receptor

ENSDARP00000068402 recombination activating gene 2

ENSDARP00000094733

ENSDARP00000068096

ENSDARP00000096678

ENSDARP00000047441 BCL6 co-repressor

ENSDARP00000096444 si:dkey-121a11.3

ENSDARP00000051258

ENSDARP00000051257

ENSDARP00000084711

ENSDARP00000091593

ENSDARP00000064665 prion protein, related sequence 1

ENSDARP00000083577 chromosome 11 open reading frame 2 (H. sapiens)

ENSDARP00000063537

ENSDARP00000063536

ENSDARP00000080925 SET and MYND domain containing 4

ENSDARP00000043801 chromosome 13 open reading frame 22-like

ENSDARP00000089906 versican b

ENSDARP00000003376 versican b

ENSDARP00000007995

ENSDARP00000019404

ENSDARP00000083396

ENSDARP00000060282 G protein-coupled receptor 19

ENSDARP00000075739

ENSDARP00000045778 death inducer-obliterator 1

ENSDARP00000066018 death inducer-obliterator 1

ENSDARP00000069339

ENSDARP00000003366

ENSDARP00000067607 zgc:136374

ENSDARP00000092069

ENSDARP00000090124 zgc:152953

ENSDARP00000014351 zgc:85716

ENSDARP00000024683 zinc finger and BTB domain containing 22

ENSDARP00000076052 si:dkey-204a24.2

ENSDARP00000052914

ENSDARP00000082769 SET domain, bifurcated 1a

ENSDARP00000095391

ENSDARP00000072076

ENSDARP00000070132

ENSDARP00000017782

ENSDARP00000053821

ENSDARP00000074443

ENSDARP00000092362

ENSDARP00000091646 zgc:153721

ENSDARP00000060468 gem (nuclear organelle) associated protein 4

ENSDARP00000025359

ENSDARP00000096890

ENSDARP00000096100

ENSDARP00000082953

ENSDARP00000008507

ENSDARP00000091800

ENSDARP00000084286

ENSDARP00000091564

ENSDARP00000091231

ENSDARP00000018100 splicing factor 3b, subunit 3

ENSDARP00000084879 si:ch211-218c6.1

ENSDARP00000089452

ENSDARP00000089451

ENSDARP00000081966

ENSDARP00000081914

ENSDARP00000089313

ENSDARP00000089311

ENSDARP00000081347

ENSDARP00000089096

ENSDARP00000061420

ENSDARP00000059651

ENSDARP00000059647

ENSDARP00000010552

ENSDARP00000074760

ENSDARP00000092541

ENSDARP00000074160 taste receptor, type 2, member 203

ENSDARP00000041875 transmembrane protein 177

ENSDARP00000072528 coagulation factor V

ENSDARP00000019686 MCM3 minichromosome maintenance deficient 3 (S. cerevisiae) associated protein

ENSDARP00000091242 MCM3 minichromosome maintenance deficient 3 (S. cerevisiae) associated protein

ENSDARP00000012786 heart of glass

ENSDARP00000071845 heart of glass

ENSDARP00000053117 collagen type XVIII, alpha 1

ENSDARP00000076760 zona pellucida protein C

ENSDARP00000096215

ENSDARP00000095543

ENSDARP00000095541

ENSDARP00000082023

ENSDARP00000079559 zgc:152894

ENSDARP00000079563 zgc:152894

ENSDARP00000029477

ENSDARP00000010305 zgc:100799

ENSDARP00000015475 toll-like receptor 18

ENSDARP00000072759 zgc:153215

ENSDARP00000093989 adenosine deaminase, RNA-specific

ENSDARP00000015422 adenosine deaminase, RNA-specific

ENSDARP00000058495

ENSDARP00000014440 zgc:55958

ENSDARP00000084654 zgc:153966

ENSDARP00000026509

ENSDARP00000057587 si:ch211-101n13.4

ENSDARP00000057585 si:ch211-101n13.4

ENSDARP00000070245

ENSDARP00000048738 tripartite motif-containing 9

ENSDARP00000022481 transforming, acidic coiled-coil containing protein 3

ENSDARP00000069816

ENSDARP00000017721 mutS homolog 6 (E. coli)

ENSDARP00000074242 zgc:55781

ENSDARP00000090621

ENSDARP00000078952

ENSDARP00000038824

ENSDARP00000094420 F-box and leucine-rich repeat protein 18

ENSDARP00000034030 F-box and leucine-rich repeat protein 18

ENSDARP00000083366

ENSDARP00000082092 zgc:153456

ENSDARP00000026079 guanylate cyclase 1, soluble, alpha 3

ENSDARP00000080083

ENSDARP00000031386 zgc:152851

ENSDARP00000091403

ENSDARP00000075886

ENSDARP00000083707 mitogen-activated protein kinase kinase kinase 7 interacting protein 3 like

ENSDARP00000072625 transmembrane protein 115

ENSDARP00000072623 transmembrane protein 115

ENSDARP00000066045

ENSDARP00000061931 alpha thalassemia/mental retardation syndrome X-linked homolog (human)

ENSDARP00000096994

ENSDARP00000095456 mitogen-activated protein kinase kinase kinase 7 interacting protein 2

ENSDARP00000019647 mitogen-activated protein kinase kinase kinase 7 interacting protein 2

ENSDARP00000013865 BTB (POZ) domain containing 7

ENSDARP00000072535

ENSDARP00000063136

ENSDARP00000002535 establishment of cohesion 1 homolog 2 (S. cerevisiae)

ENSDARP00000037368 si:dkeyp-93d12.1

ENSDARP00000011821

ENSDARP00000058027

ENSDARP00000041548 zinc finger CCCH-type containing 10

ENSDARP00000058642 zinc finger protein 750

ENSDARP00000075118 zgc:101877

ENSDARP00000040064

ENSDARP00000094585

ENSDARP00000094512

ENSDARP00000055239 periplakin

ENSDARP00000077459 G protein-coupled receptor 146

ENSDARP00000081928 zgc:153228

ENSDARP00000052347 exosome component 6

ENSDARP00000070860 zgc:123259

ENSDARP00000068304

ENSDARP00000080440

ENSDARP00000056892

ENSDARP00000012447

ENSDARP00000051544

ENSDARP00000051543

ENSDARP00000032320

ENSDARP00000073786

ENSDARP00000069554

ENSDARP00000009684

ENSDARP00000038181

Single-copy genes with lengths of exon from 700bp to 800 bp

ENSDARP00000052748

ENSDARP00000077653

ENSDARP00000077651

ENSDARP00000077650

ENSDARP00000076957

ENSDARP00000033038

ENSDARP00000017287 prion protein, related sequence 3

ENSDARP00000025618 kelch-like 15 (Drosophila)

ENSDARP00000073495

ENSDARP00000066857 zgc:85973

ENSDARP00000083528 zgc:153363

ENSDARP00000058410 holocarboxylase synthetase (biotin-(proprionyl-Coenzyme A-carboxylase (ATP-hydrolysing)) ligase)

ENSDARP00000067377

ENSDARP00000091044

ENSDARP00000094726 death inducer-obliterator 1

ENSDARP00000096055

ENSDARP00000020607

ENSDARP00000092359

ENSDARP00000082958

ENSDARP00000062359 nucleoporin 205

ENSDARP00000062215 nucleoporin 205

ENSDARP00000084512 si:dkey-25f3.3

ENSDARP00000011566 solute carrier family 33 (acetyl-CoA transporter), member 1

ENSDARP00000072514 G protein-coupled receptor 161

ENSDARP00000094999

ENSDARP00000080583

ENSDARP00000078783 zgc:158466

ENSDARP00000094778 death effector domain-containing 1

ENSDARP00000094012 FAD1 flavin adenine dinucleotide synthetase homolog (S. cerevisiae)

ENSDARP00000080453 zgc:158219

ENSDARP00000029449

ENSDARP00000068202

ENSDARP00000089706 prostaglandin E synthase 2-like

ENSDARP00000054416 zgc:113229

ENSDARP00000096943 zgc:113229

ENSDARP00000010075 mediator complex subunit 7

ENSDARP00000026819 zgc:152769

ENSDARP00000048163 phosphoribosylaminoimidazole carboxylase, phosphoribosylaminoimidazole succinocarboxamide synthetase

ENSDARP00000093425 zgc:103638

ENSDARP00000062847 si:dkey-30j22

Single-copy genes with lengths of exon from 600bp to 700bp

ENSDARP00000089700

ENSDARP00000057897

ENSDARP00000089697

ENSDARP00000087727

ENSDARP00000067055

ENSDARP00000066854

ENSDARP00000056680

ENSDARP00000076519 wu:fb02f03

ENSDARP00000096461 zgc:92337

ENSDARP00000062735 zgc:92337

ENSDARP00000016572 exportin 7

ENSDARP00000013436 N-acyl phosphatidylethanolamine phospholipase D

ENSDARP00000084180

ENSDARP00000065406 hypoxia-inducible factor 1, alpha subunit, like 2

ENSDARP00000030178 si:dkey-98n4.1

ENSDARP00000045795 si:dkey-98n4.1

ENSDARP00000082851

ENSDARP00000028244 protein phosphatase 1, regulatory subunit 10

ENSDARP00000043553 protein phosphatase 1, regulatory subunit 10

ENSDARP00000086332

ENSDARP00000053587 zgc:112481

ENSDARP00000092937 intraflagellar transport 80 homolog (Chlamydomonas)

ENSDARP00000075402 zgc:113279

ENSDARP00000075551 zgc:113279

ENSDARP00000062675 suppressor of Ty 5 homolog (S. cerevisiae)

ENSDARP00000062673 suppressor of Ty 5 homolog (S. cerevisiae)

ENSDARP00000014434 sterile alpha and TIR motif containing 1

ENSDARP00000053002

ENSDARP00000096636 X-ray repair complementing defective repair in Chinese hamster cells 6

ENSDARP00000096634 X-ray repair complementing defective repair in Chinese hamster cells 6

ENSDARP00000062519

ENSDARP00000092066

ENSDARP00000042495

ENSDARP00000072160 Leo1, Paf1/RNA polymerase II complex component, homolog (S. cerevisiae)

ENSDARP00000076825 transmembrane protein 37

ENSDARP00000059661

ENSDARP00000039212 zgc:158327

ENSDARP00000071911

ENSDARP00000080636

ENSDARP00000070753

ENSDARP00000070224

ENSDARP00000043865 polo-like kinase 4 (Drosophila)

ENSDARP00000078791 zgc:158466

ENSDARP00000021873 zgc:63579

ENSDARP00000028101

ENSDARP00000059052 ribonuclease P/MRP 38 subunit

ENSDARP00000005597 zgc:100846

ENSDARP00000057755 zgc:100846

ENSDARP00000060993 askopos

ENSDARP00000089981

ENSDARP00000056543

ENSDARP00000024667 microsomal triglyceride transfer protein

ENSDARP00000093616 zinc finger, CCHC domain containing 7- like

ENSDARP00000038514 zinc finger, CCHC domain containing 7- like

ENSDARP00000092516 GRB2-associated binding protein 1

ENSDARP00000067667 zgc:65891

ENSDARP00000008228 nei endonuclease VIII-like 3 (E. coli)

ENSDARP00000063735 si:dkeyp-72h1.2

ENSDARP00000025022

ENSDARP00000061842 si:dkey-65b13.2

ENSDARP00000078901

ENSDARP00000068895 zgc:153648

ENSDARP00000093158 endothelium-specific receptor tyrosine kinase 1

ENSDARP00000057296 myeloproliferative leukemia virus oncogene, like

ENSDARP00000042367 fumarylacetoacetate hydrolase domain containing 1

ENSDARP00000071960 zgc:162544

ENSDARP00000092154

ENSDARP00000071604 zgc:113305

ENSDARP00000091530 zgc:163091

ENSDARP00000025759

ENSDARP00000051553

ENSDARP00000051269

ENSDARP00000051296

ENSDARP00000051268

ENSDARP00000003259 zgc:112042

Single-copy genes with lengths of exon from 500bp to 600bp

ENSDARP00000016813 zinc finger, RAN-binding domain containing 3

ENSDARP00000027775 zgc:85683

ENSDARP00000067530 synapsin IIa

ENSDARP00000029798

ENSDARP00000058661 dihydrolipoamide branched chain transacylase E2

ENSDARP00000083967 chromatin assembly factor 1, subunit A (p150)

ENSDARP00000096357 si:ch211-129c21.6

ENSDARP00000072407 microtubule associated serine/threonine kinase-like

ENSDARP00000066648 polymerase (DNA directed), alpha 1

ENSDARP00000066632 polymerase (DNA directed), alpha 1

ENSDARP00000073791

ENSDARP00000076732 zgc:113274

ENSDARP00000065035 phosphorylated adaptor for RNA export

ENSDARP00000063842 peptidyl-tRNA hydrolase 2

ENSDARP00000090764

ENSDARP00000004330

ENSDARP00000038850

ENSDARP00000012780

ENSDARP00000090061 tripartite motif-containing 24

ENSDARP00000030077 zinc finger, CCCH-type with G patch domain

ENSDARP00000033739

ENSDARP00000031774 methyl-CpG binding domain protein 1

ENSDARP00000083233 zgc:136566

ENSDARP00000093035 spectrin alpha 2

ENSDARP00000093032 spectrin alpha 2

ENSDARP00000093029 spectrin alpha 2

ENSDARP00000093027 spectrin alpha 2

ENSDARP00000093025 spectrin alpha 2

ENSDARP00000093023 spectrin alpha 2

ENSDARP00000065555 si:rp71-1p14.5

ENSDARP00000091993 zgc:136279

ENSDARP00000018567 protein phosphatase methylesterase 1

ENSDARP00000091486 hypoxia-inducible factor 1, alpha subunit, like 2

ENSDARP00000051769 brain protein 16

ENSDARP00000095875

ENSDARP00000076622

ENSDARP00000095393

ENSDARP00000053875

ENSDARP00000049372 FtsJ methyltransferase domain containing 2

ENSDARP00000087582

ENSDARP00000018333

ENSDARP00000091456 excision repair cross-complementing rodent repair deficiency, complementation group 2

ENSDARP00000027008 excision repair cross-complementing rodent repair deficiency, complementation group 2

ENSDARP00000016166

ENSDARP00000042520

ENSDARP00000053760 BMS1-like, ribosome assembly protein (yeast)

ENSDARP00000026695 kinesin family member 23

ENSDARP00000060999 P

ENSDARP00000013949 zgc:114141

ENSDARP00000030569

ENSDARP00000011892 protein phosphatase 1, regulatory (inhibitor) subunit 13 like

ENSDARP00000026588 ubiquitin specific peptidase 9

ENSDARP00000027333

ENSDARP00000073017 zgc:77397

ENSDARP00000095942 zgc:63779

ENSDARP00000063571 zgc:56388

ENSDARP00000039745 erythrocyte membrane protein band 4.1 (elliptocytosis 1, RH-linked)

ENSDARP00000072745 testis expressed 10

ENSDARP00000058619 zgc:66432

ENSDARP00000093573

ENSDARP00000033960

ENSDARP00000059369

ENSDARP00000010838

ENSDARP00000034283 zgc:113197

ENSDARP00000090320

ENSDARP00000029765 zgc:66427

ENSDARP00000014151 zinc finger protein 622

ENSDARP00000084087

ENSDARP00000084104

ENSDARP00000028918

ENSDARP00000071242 im:7151414

ENSDARP00000093388 im:7151414

ENSDARP00000054183 zgc:123172

ENSDARP00000057879 polymerase (DNA directed), lambda

ENSDARP00000069175

ENSDARP00000078271

ENSDARP00000090524

ENSDARP00000062374

ENSDARP00000020705 zgc:92666

ENSDARP00000079535 zgc:92666

ENSDARP00000070580 zgc:63767

ENSDARP00000001475 nuclear receptor co-repressor 2

ENSDARP00000023459

ENSDARP00000073865 nudix (nucleoside diphosphate linked moiety X)-type motif 22

ENSDARP00000037730 hyaluronan mediated motility receptor

ENSDARP00000078554

ENSDARP00000018762

ENSDARP00000036002 tumor necrosis factor receptor superfamily, member 21

ENSDARP00000032296 si:ch211-169j21.3

ENSDARP00000005919 si:ch211-169j21.3

ENSDARP00000061167

ENSDARP00000090386

ENSDARP00000061671 PRP39 pre-mRNA processing factor 39 homolog (yeast)

ENSDARP00000017601

ENSDARP00000065237

ENSDARP00000032020

ENSDARP00000056394

ENSDARP00000034889 nuclear autoantigenic sperm protein (histone-binding)

ENSDARP00000057284 nuclear autoantigenic sperm protein (histone-binding)

ENSDARP00000081852 zgc:153216

ENSDARP00000071703 asparagine-linked glycosylation 1 homolog (yeast, beta-1,4-mannosyltransferase)

ENSDARP00000094383 asparagine-linked glycosylation 1 homolog (yeast, beta-1,4-mannosyltransferase)

ENSDARP00000087122

ENSDARP00000071352

ENSDARP00000073498 coatomer protein complex, subunit beta 1

ENSDARP00000069932

ENSDARP00000004461 SAFB-like, transcription modulator

ENSDARP00000007066 SAFB-like, transcription modulator

ENSDARP00000077369

ENSDARP00000089274 zgc:153242

ENSDARP00000068049

ENSDARP00000077215

ENSDARP00000083539

ENSDARP00000027320 zgc:103692

ENSDARP00000088441 zgc:158432

ENSDARP00000077902 zgc:152897

ENSDARP00000004735 cyclin H

ENSDARP00000050911 peroxisomal biogenesis factor 12

ENSDARP00000050910 peroxisomal biogenesis factor 12

ENSDARP00000077314

ENSDARP00000077307

ENSDARP00000051830 zgc:152816
