# Supplementary material for: Phylogenomic Analysis Resolves the Formerly Intractable Adaptive Diversification of the Endemic Clade of East Asian Cyprinidae (Cypriniformes)
Source: PLoS One. 2010 Oct 20;5(10):e13508. doi: 10.1371/journal.pone.0013508 (PMC2958143; doi:10.1371/journal.pone.0013508)
Supplement: Appendix S4 — The detailed information for each of the 100 loci sampled in the present study. (0.29 MB DOC) [file pone.0013508.s004.doc]

Additional data file 4

The detailed information for each of the 100 loci sampled in the present study, including aligned length excluding ambiguous regions (L), proportions of exon and GC content, ratio of transistion and transversion (ti/tv), mean pairwise divergence (p-distance) (MPD) and percent variable (VS) and parsimony informative sites (IS) among all taxa and within the endemic East-Asian cyprinids, respectively. Accession numbers of *Danio rerio* are from Ensembl and GenBank database.

| Gene name | Length  L | exon | Ti/tv | GC% | MPD | VS | IS | E  MPD | E  VS | | E  IS | *D. rerio*  accession numbers |
| --- | --- | --- | --- | --- | --- | --- | --- | --- | --- | --- | --- | --- |
| Ptr (si:ch211-105n9.1), hypothetical protein LOC564097 | 732 | 100% | 3.214 | 46.20% | 0.033 | 13.40% | 5.20% | 0.2370 | 8.47% | | 3.83% | ENSDARE00000145053 |
| T-box 1, brain (tbr1), | 675 | 100% | 1.558 | 60.30% | 0.0093 | 5.04% | 0.74% | 0.0044 | 2.37% | | 0.15% | ENSDARE00000055502 |
| early growth response 2B | 804 | 100% | 2.231 | 60.90% | 0.0235 | 10.95% | 2.49% | 0.0138 | 6.09% | | 1.00% | AC144827 |
| rhodopsin | 864 | 100% | 1.759 | 56.90% | 0.0586 | 16.42% | 4.35% | 0.0368 | 11.23% | | 5.90% | BC164171 |
| large subunit ribosomal RNA gene | 597 | 0 | 1.396 | 71.52% | 0.0325 | 16.70% | 4.59% | 0.0098 | 11.82% | | 2.47% | EF417169 |
| myosin, heavy polypeptide 6 | 747 | 100% | 3.185 | 46.27% | 0.0454 | 20.21% | 6.02% | 0.0274 | 10.84% | | 3.48% | AY138982 |
| Recombination activating gene 2 sequence | 1248 | 100% | 2.468 | 47.50% | 0.0399 | 17.71% | 4.89% | 0.0240 | 10.90% | | 2.56% | NM_131385 |
| sorting nexin 33 | 714 | 100% | 4.601 | 55.42% | 0.04 | 18.63% | 4.90% | 0.0225 | 8.82% | | 3.36% | BC169193 |
| ectodermal-neural cortex 1-like protein (ENC1) gene | 807 | 100% | 3.587 | 55.70% | 0.0339 | 14.25% | 4.71% | 0.0245 | 10.29% | | 2.60% | EF032975 |
| glycosyltransferase | 807 | 100% | 2.578 | 47.70% | 0.0359 | 16.97% | 3.03% | 0.0223 | 10.67% | | 2.06% | EF032988 |
| beta-actin gene | 401 | 74.1% | 1.063 | 53.45% | 0.099 | 16.06% | 5.62% | 0.047 | 10.44% | | 4.82% | BC045879 |
| homeodomain protein  C1a | 525 | 100% | 1.212 | 55.40% | 0.0321 | 15.62% | 3.81% | 0.020 | 8.76% | | 1.91% | [AJ306432](http://www.ncbi.nlm.nih.gov/nuccore/13397816?ordinalpos=1&itool=EntrezSystem2.PEntrez.Sequence.Sequence_ResultsPanel.Sequence_RVDocSum) |
| Recombination activating gene 1 | 1503 | 100% | 2.636 | 50.30% | 0.0365 | 16.73% | 3.92% | 0.0251 | 10.69% | | 2.66% | [NM_131389](http://www.ncbi.nlm.nih.gov/nuccore/18859302?ordinalpos=1&itool=EntrezSystem2.PEntrez.Sequence.Sequence_ResultsPanel.Sequence_RVDocSum) |
| patched domain-containing protein 1, partial cds | 819 | 100% | 4.337 | 46.36% | 0.0312 | 12.82% | 4.76% | 0.0219 | 7.57% | | 3.54% | NM_001030185 |
| zinc finger and BTB domain containing 22, transcript variant 1 (zbtb22) | 816 | 100% | 1.963 | 55.80% | 0.0612 | 26.29% | 7.23% | 0.0473 | 19.61% | | 5.76% | XM_684451 |
| homeodomain protein  A9a | 1112 | 59.9% | 1.187 | 46.90% | 0.0508 | 18.12% | 2.60% | 0.029 | 12.02% | | 1.97% | NM_131532 |
| S7 ribosomal protein gene, intron 1 | 935 | 0 | 0.803 | 34.80% | 0.1521 | 44.28% | 10.70% | 0.0875 | 31.02% | | 7.81% | AY325795 |
| homeodomain protein  D9a | 582 | 100% | 1.823 | 28.14% | 0.0645 | 5.33% | 3.27% | 0.0205 | 8.25% | | 2.41% | NM_001128296 |
| adenylosuccinate lyase part1 | 420 | 36.5% | 1.371 | 47.55% | 0.0527 | 14.76% | 0.95% | 0.0528 | 7.60% | | 0.49% | [NM_199899](http://www.ncbi.nlm.nih.gov/nuccore/41054024?ordinalpos=1&itool=EntrezSystem2.PEntrez.Sequence.Sequence_ResultsPanel.Sequence_RVDocSum) |
| c-Jun | 603 | 100% | 0.941 | 62.35% | 0.0341 | 15.76% | 3.32% | 0.0197 | 7.96% | | 2.16% | DQ003340 |
| caudal type homeo box transcription factor 4 | 703 | 33.7% | 1.046 | 28.88% | 0.0647 | 28.88% | 8.82% | 0.0571 | 23.47% | | 7.26% | BC160643 |
| adenylosuccinate lyase part1 | 345 | 42.6% | 1.871 | 45.80% | 0.0407 | 20.29% | 11.01% | 0.0529 | | 17.68% | 9.86% | BC045891 |
| Gastrulation brain homeobox 1 (gbx1) | 793 | 31.4% | 1.609 | 41.90% | 0.0411 | 21.48% | 5.28% | 0.0289 | 13.07% | | 3.27% | AF288763 |
| luteinizing hormone | 702 | 100% | 1.975 | 55.65% | 0.0675 | 24.79% | 7.98% | 0.0455 | 14.96% | | 5.41% | AY714133 |
| kelch-like 11 | 711 | 100% | 3.907 | 49.30% | 0.0357 | 12.52% | 3.37% | 0.0202 | 6.47% | | 1.97% | BC045881 |
| clone PMB18-T RH | 1025 | 0 | 1.343 | 42.12% | 0.0292 | 5.56% | 0.78% | 0.0292 |  | |  |  |
| homeodomain protein  A13a | 450 | 100% | 2.049 | 56.60% | 0.0398 | 15.11% | 2.44% | 0.0170 | 8.33% | | 1.78% | BC133882 |
| homeodomain protein  B5b | 1161 | 58.1% | 0.801 | 43.54% | 0.0289 | 15.21% | 2.23% | 0.0156 | 8.33% | | 1.17% | BC078285 |
| inhibin, beta B | 573 | 100% | 2.917 | 57.90% | 0.0345 | 15.53% | 4.54% | 0.0223 | 9.95% | | 2.27% | BC162143 |
| sphingosine 1-phosphate receptor (edg1) | 651 | 100% | 2.681 | 55.30% | 0.0448 | 17.51% | 6.30% | 0.0302 | 10.91% | | 4.30% | [AF321294](http://www.ncbi.nlm.nih.gov/nuccore/12018161?ordinalpos=1&itool=EntrezSystem2.PEntrez.Sequence.Sequence_ResultsPanel.Sequence_RVDocSum) |
| homeodomain protein  A2b | 1561 | 52.3% | 1.501 | 46.38% | 0.0116 | 7.687% | 1.28% | 0.0286 | 45.48% | | 0.09% | [NM_131106](http://www.ncbi.nlm.nih.gov/nuccore/18858824?ordinalpos=1&itool=EntrezSystem2.PEntrez.Sequence.Sequence_ResultsPanel.Sequence_RVDocSum) |
| homeodomain protein  D4a | 812 | 50.2% | 1.204 | 46.94% | 0.0245 | 23.65% | 5.91% | 0.025 | 15.52% | | 4.54% | AY168642 |
| somatostatin receptor 2 | 972 | 100% | 2.846 | 51.35% | 0.0502 | 18.83% | 3.70% | 0.027 | 8.54% | | 2.47% | XM_689793 |
| dermatan4 sulfotransferase 1 | 621 | 100% | 3.165 | 46.10% | 0.0439 | 16.91% | 2.74% | 0.020 | 6.76% | | 1.45% | BC115151 |
| homeodomain b6b | 373 | 65% | 1.529 | 48.91% | 0.0211 | 20.55% | 4.85% | 0.0288 | 18.48% | | 2.77% | AF071254 |
| alpha-1-microglobulin  /bikunin | 37 | 100% | 1.5 | 48.92% | 0.0515 | 15.20% | 0.76% | 0.0258 | 5.30% | | 0 | BC165143.1 |
| opsin 1 | 306 | 100% | 2.076 | 45.40% | 0.0413 | 15.03% | 4.90% | 0.0286 | 8.17% | | 4.58% | BC075989 |
| gonadotropin alpha subunit | 627 | 11.5% | 0.137 | 33.00% | 0.0842 | 28.90% | 5.046% | 0.0817 | 28.44% | | 5.05% | AY522553 |
| homeodomain protein  B1b | 603 | 100% | 1.196 | 49.90% | 0.080 | 12.94% | 2.82% | 0.0770 | 6.63% | | 0.66% | BC133876 |
| homeodomain protein  B1a | 836 | 68.6% | 0.93 | 51.63% | 0.0439 | 27.06% | 6.79% | 0.0317 | 21.10% | | 4.77% | [NM_131115](http://www.ncbi.nlm.nih.gov/nuccore/18858828?ordinalpos=1&itool=EntrezSystem2.PEntrez.Sequence.Sequence_ResultsPanel.Sequence_RVDocSum) |
| homeodomain protein  B3a | 690 | 100% | 5.733 | 54.02% | 0.0224 | 9.71% | 3.63% | 0.0172 | 7.39% | | 3.62% | NM_131117 |
| kelch repeat and BTB (POZ) domain | 504 | 100% | 4.347 | 52.98% | 0.0548 | 22.82% | 7.94% | 0.0345 | 14.09% | | 4.56% | BC054912 |
| V1r pheromone receptor-like | 795 | 100% | 2.22 | 64.50% | 0.0350 | 16.60% | 3.65% | 0.0197 | 8.55% | | 2.52% | AY900118 |
| Casf2 | 562 | 0 | 1.908 | 43.90% |  |  |  | 0.0999 | 22.95% | | 2.85% |  |
| zinc finger and BTB domain | 714 | 100% | 1.55 | 54.83% | 0.1152 | 30.39% | 19.89% | 0.1025 | 24.37% | | 18.63% | XM_684451 |
| connexin 52.6 | 1053 | 0 | 2.96 | 51.21% | 0.0507 | 19.66% | 3.61% | 0.0247 | 7.60% | | 5.70% | DQ133441 |
| homeodomain protein  c12b | 978 | 77.9% | 1.576 | 44.22% | 0.0720 | 23.98% | 3.70% | 0.0354 | 6.76% | | 0.082% | BC162850 |
| homeodomain protein  c12a | 628 | 23.4% | 0.969 | 43.10% | 0.0231 | 17.83% | 1.43% | 0.0242 | 15.92% | | 1.15% | BC162872 |
| interphotoreceptor retinoid-binding protein | 822 | 100% | 1.65 | 48.70% | 0.0580 | 21.25% | 8.30% | 0.0427 | 18.68% | | 5.13% | BC060944 |
| early growth response 3 | 912 | 100% | 4.042 | 58.33% | 0.0314 | 12.71% | 3.51% | 0.0225 | 7.02% | | 1.86% | EU409788 |
| early growth response 1 | 804 | 100% | 2.232 | 60.90% | 0.0226 | 10.95% | 2.49% | 0.0136 | 6.09% | | 0.99% | NM_131248 |
| one-eyed pinhead | 760 | 0 | 1.903 | 39.10% | 0.0496 | 24.21% | 5.00% | 0.0507 | 21.71% | | 5.00% | BC152235 |
| apoa1 | 492 | 0 | 1.074 | 49.60% | 0.1191 | 29.09% | 11.10% | 0.0784 | 20.81% | | 10.91% | AJ245491 |
| apolipoprotein A-I-2 | 327 | 0 | 0.749 | 51.68% |  |  |  | 0.2142 | 51.68% | | 30.88% |  |
| periplakin | 990 | 0 | 1.463 | 45.53% | 0.0403 | 20.40% | 4.747% | 0.0278 | 15.12% | | 3.63% | NM_001137666 |
| homeodomain protein  A1a | 864 | 82.8% | 1.889 | 47.60% | 0.0462 | 21.36% | 4.50% | 0.0315 | 14.08% | | 3.45% | [NM_131536](http://www.ncbi.nlm.nih.gov/nuccore/18858822?ordinalpos=1&itool=EntrezSystem2.PEntrez.Sequence.Sequence_ResultsPanel.Sequence_RVDocSum) |
| homeodomain protein  C4a | 1089 | 50.4% | 1.695 | 48.25% | 0.0093 | 4.13% | 0.64% | 0.0062 | 2.39% | | 0.551% | AF071264 |
| homeodomain protein  C8a | 851 | 45% | 1.224 | 44.70% | 0.0534 | 23.42% | 3.40% | 0.0312 | 12.30% | | 2.22% | [BC165923](http://www.ncbi.nlm.nih.gov/nuccore/182890003?ordinalpos=1&itool=EntrezSystem2.PEntrez.Sequence.Sequence_ResultsPanel.Sequence_RVDocSum) |
| Casf1 | 642 | 100% | 1.538 | 46.50% | 0.0840 | 19.78% | 2.18% | 0.0473 | 9.50% | | 0.16% | XM_684005.2 |
| Trim9 | 322 | 0 | 0.993 | 46.09% |  |  |  | 0.1076 | 25.46% | | 9.03% |  |
| homeodomain protein  A5a1 | 998 | 483  48.4% | 1.151 | 43.92% | 0.0330 | 18.34% | 3.707% | 0.0379 | 13.73% | | 2.31% | BC162975 |
| homeodomain protein  c13a | 573 | 100% | 0.999 | 52.60% | 0.0317 | 16.06% | 1.75% | 0.027 | 12.39% | | 1.57% | [NM_131543](http://www.ncbi.nlm.nih.gov/nuccore/50233776?ordinalpos=1&itool=EntrezSystem2.PEntrez.Sequence.Sequence_ResultsPanel.Sequence_RVDocSum) |
| melanocortin 4 receptor | 894 | 100% | 2.448 | 51.60% | 0.0366 | 16.67% | 4.25% | 0.0225 | 9.06% | | 2.57% | AY161850 |
| interphotoreceptor  (IRBP1) | 303 | 100% | 1.068 | 47.30% | 0.0953 | 53.80% | 4.29% | 0.0223 | 10.56% | | 1.98% | BC060944 |
| alpha-1D adrenoreceptor | 441 | 100% | 1.71 | 57.00% | 0.0463 | 18.37% | 5.67% | 0.0305 | 11.79% | | 2.72% | XM_691951 |
| amylase, alpha 2A | 231 | 100% | 1.385 | 43.06% |  |  |  | 0.0515 | 18.11% | | 7.17% | BC062867 |
| wingless-type MMTV integration site family | 775 | 30.6% | 1.111 | 47.50% | 0.0291 | 12.47% | 1.80% | 0.0236 | 10.67% | | 1.67% | BX548172.7 |
| dermacan | 369 | 100% | 1.706 | 47.23% | 0.0607 | 28.83% | 6.78% | 0.0341 | 13.28% | | 4.34% | AB119257 |
| gonadotropin alpha subunit | 683 | 27.2% | 0.682 | 35.18% | 0.0426 | 17.42% | 3.95% | 0.0464 | 14.20% | | 3.95% | AY522553 |
| alpha-1D adrenoreceptor3 | 444 | 100% | 1.509 | 56.60% | 0.0497 | 20.50% | 5.63% | 0.0358 | 13.96% | | 3.83% | XM_691951 |
| mediator complex subunit 7 | 480 | 100% | 1.233 | 50.60% | 0.0369 | 14.38% | 5.21% | 0.0298 | 10.42% | | 4.17% | BC046018 |
| growth hormone | 713 | 27.8% | 0.766 | 37.00% | 0.0204 | 28.75% | 2.03% | 0.0502 | 28.47% | | 5.75% | AJ937858 |
| Tbx21 tbet | 735 | 100% | 2.328 | 59.70% | 0.0695 | 26.67% | 7.22% | 0.046 | 15.92% | | 5.17% | XM_001338226 |
| homeodomain protein b9a | 192 | 100% | 1.08 | 48.02% | 0.0525 | 20% | 5.64% | 0.0492 | 13.85% | | 1.03% | AF071256.1 |
| homeodomain protein  hoxD3a | 1201 | 83.9% | 2.608 | 49.66% | 0.0335 | 12.49% | 2.33% | 0.0179 | 8.16% | | 0.92% | NM_131125.1 |
| homeodomain protein  hoxD12a | 736 | 64.4% | 1.374 | 47.89% | 0.0347 | 12.91% | 3.13% | 0.0226 | 7.80% | | 2.99% | NM_001126486 |
| homeodomain protein  hoxC9a | 477 | 100% | 1.934 | 56.20% | 0.0151 | 6.29% | 1.68% | 0.0097 | 2.43% | | 1.47% | BC164046.1 |
| homeodomain protein  B8a | 1304 | 40.7% | 1.62 | 47.66% | 0.0177 | 13.96% | 4.91% | 0.0345 | 12.58% | | 4.14% | BC053287 |
| cellular myelocytomatosis cmyc | 2275 | 53.1% | 1.969 | 52.07% | 0.0366 | 17.00% | 4.09% | 0.0283 | 8.97% | | 2.64% | BC053281 |
| S6 kinase 1 (S6K1) gene | 889 | 12.5% | 1.295 | 42.90% | 0.089 | 25.76% | 5.34% | 0.0583 | 15.64% | | 4.05% | EF373681 |
| homeodomain protein  B13a | 408 | 100% | 2.235 | 55.08% | 0.0326 | 10.71% | 5.35% | 0.0251 | 9.0% | | 1.46% | BC117625 |
| homeodomain protein  B13a-s | 51 | 100% | 1.005 | 52.45% | 0.0490 | 17.64% | 7.84% | 0.0285 | 7.84% | | 3.92% | BC117625 |
| homeodomain protein  B5a | 570 | 100% | 0.721 | 54.30% | 0.0418 | 17.45% | 7.68% | 0.0416 | 17.45% | | 5.76% | BC071493 |
| homeodomain protein  B8b | 862 | 66.7% | 2.092 | 48.02% | 0.0231 | 10.789% | 2.32% | 0.0194 | 8.59% | | 1.74% | BC162167 |
| myogenic differentiation 1 | 821 | 58.8% | 1.806 | 46.08% | 0.0277 | 19.65% | 6.26% | 0.0412 | 17.17% | | 5.36% | BC114261 |
| 28S ribosomal RNA gene | 747 | 0 | 0.498 | 50.34% |  |  |  | 0.0189 | 4.82% | | 0.54% |  |
| homeodomain protein  D11a | 357 | 100% | 1.896 | 43.23% | 0.0254 | 10.64% | 3.64% | 0.0164 | 5.32% | | 3.08% | BC114271 |
| homeodomain protein  D10a-2 92 | 402 | 100% | 1.448 | 48.47% | 0.0123 | 7.21% | 1.99% | 0.0094 | 4.98% | | 1.74% | BC071329 |
| homeodomain protein  c11 | 177 | 100% | 1.424 | 37.47% | 0.0141 | 5.65% | 1.70% | 0.011 | 3.98% | | 1.70% | BC164748 |
| ryanodine receptor 3 | 888 | 100% | 3.423 | 47.04% | 0.0373 | 16.89% | 4.84% | 0.0241 | 9.23% | | 3.83% | XM_001922078 |
| insulin receptor a | 1501 | 22.4% | 0.734 | 32.70% | 0.1253 | 20.45% | 3.13% | 0.0788 | 15.99% | | 2.99% | AF400271.1 |
| insulin receptor b | 614 | 49.8% | 1.426 | 45.54% | 0.0572 | 19.45% | 6.321% | 0.0557 |  | |  | AF400272 |
| connective tissue growth factor | 1731 | 52.9% | 1.365 | 46.87% | 0.0693 | 19.30% | 3.41% | 0.0413 | 9.99% | | 2.37% | EF524114.1 |
| SREB2 | 756 | 100% | 1.957 | 60.67% | 0.0391 | 15.02% | 3.03% | 0.0339 | 10.80% | | 2.64% | AB040805.1 |
| pleomorphic adenoma gene-like 2 | 840 | 100% | 2.83 | 54.50% | 0.0219 | 10.12% | 2.14% | 0.0140 | 6.31% | | 1.19% | AF186476 |
| actin, alpha 1, skeletal muscle | 401 | 70.9% | 2.199 | 51.70% | 0.0165 | 10.47% | 2.24% | 0.0193 | 8.98% | | 2.24% | BC168467.1 |
| alpha-tubulin | 91 | 100% |  | 38.89% |  |  |  |  |  | |  | emb|CU633810.6 |
| similar to pecanex homolog | 102 | 0 | 0.25 | 57.43% |  |  |  | 0.0492 | 4.90% | | 0.98% | [XM_001923960.1|](http://www.ncbi.nlm.nih.gov/entrez/query.fcgi?cmd=Retrieve&db=Nucleotide&list_uids=189527117&dopt=GenBank&RID=ZXST3HVX014&log$=nucltop&blast_rank=1) |
| manganese superoxide dismutase | 366 | 100% | 1.211 | 47.80% | 0.0465 | 10.66% | 1.91% | 0.0224 | 4.37% | | 0.82% | AY195857.1 |
| eukaryotic translation initiation factor 1B | 342 | 100% | 1.312 | 47.30% | 0.0136 | 5.56% | 0 | 0.0095 | 3.22% | | 0 | NM_199588.3 |
