# Supplementary material for: Phylogenomic Analysis Resolves the Formerly Intractable Adaptive Diversification of the Endemic Clade of East Asian Cyprinidae (Cypriniformes)
Source: PLoS One. 2010 Oct 20;5(10):e13508. doi: 10.1371/journal.pone.0013508 (PMC2958143; doi:10.1371/journal.pone.0013508)
Supplement: Appendix S5 — Chi-square tests of homogeneous base frequencies among all partition strategies. (0.05 MB DOC) [file pone.0013508.s005.doc]

Additional data file 5

Chi-square tests of homogeneous base frequencies among all partition strategies. A- *Mylopharyngodon piceus*; B**-** *Ctenopharyngodon idella*; C- *Hypophthalmichthys molitrix*; D- *Hypophthalmichthys nobilis*; E- *Squaliobarbus curriculus*；F- *Megalobrama amblycephala*； G- *Elopichthys bambusa*; H- *Xenocypris argentea*； I- *Culter alburnus*； J- *Opsariichthys bidens*；K- *Ochetobius elongatus*; L- *Luciobrama macrocephalus*; M- *Danio rerio*

| *Data set* | *GC% content* | | | | | | | | | | | | | | | |
| --- | --- | --- | --- | --- | --- | --- | --- | --- | --- | --- | --- | --- | --- | --- | --- | --- |
|  | No.of sites (bp) | A | B | C | D | E | F | G | H | I | J | K | L | M | Mean | P value^﹟^ |
| All sites | 71132 | 0.499 | 0.492 | 0.499 | 0.495 | 0.494 | 0.493 | 0.501 | 0.50 | 0.496 | 0.50 | 0.501 | 0.501 | 0.51 | 0.50 | 0.00000 |
| intron | 20047 | 0.403 | 0.380 | 0.392 | 0.395 | 0.385 | 0.381 | 0.404 | 0.403 | 0.392 | 0.383 | 0.405 | 0.379 | 0.436 | 0.394 | 0.00000 |
| exon | 51085 | 0.531 | 0.53 | 0.531 | 0.529 | 0.530 | 0.532 | 0.531 | 0.529 | 0.530 | 0.506 | 0.531 | 0.531 | 0.522 | 0.53 | 0.8059 |
| 1st codon | 17027 | 0.525 | 0.527 | 0.526 | 0.527 | 0.530 | 0.528 | 0.525 | 0.528 | 0.526 | 0.530 | 0.526 | 0.526 | 0.525 | 0.527 | 1.00000 |
| 2nd codon | 17027 | 0.437 | 0.437 | 0.442 | 0.440 | 0.436 | 0.442 | 0.438 | 0.435 | 0.441 | 0.447 | 0.439 | 0.445 | 0.438 | 0.440 | 0.996 |
| 3rd codon | 17027 | 0.63 | 0.624 | 0.625 | 0.622 | 0.624 | 0.626 | 0.629 | 0.626 | 0.623 | 0.631 | 0.628 | 0.623 | 0.603 | 0.627 | 0.0823 |
| Intron+  3rd codon | 37074 | 0.517 | 0.502 | 0.513 | 0.508 | 0.505 | 0.501 | 0.520 | 0.519 | 0.510 | 0.512 | 0.521 | 0.518 | 0.551 | 0.514 | 0.00000 |

^﹟^Pairwise χ^2^ tests.
