# Supplementary material for: Phylogenomic Analysis Resolves the Formerly Intractable Adaptive Diversification of the Endemic Clade of East Asian Cyprinidae (Cypriniformes)
Source: PLoS One. 2010 Oct 20;5(10):e13508. doi: 10.1371/journal.pone.0013508 (PMC2958143; doi:10.1371/journal.pone.0013508)
Supplement: Appendix S6 — Statistical comparisons of alternative topologies, including the combined dataset and previous evolutionary hypothesis using approximately AU test, SH test, KH test and WKH test. (0.03 MB DOC) [file pone.0013508.s006.doc]

Additional data file 6

Statistical comparisons of alternative topologies, including the combined dataset and previous evolutionary hypothesis using approximately AU test, SH test, KH test and WKH test.

| Topology | -ln L | -Ln L Diff | AU test: P | SH test: P | KH test: P | WSH test: P | KH test: P | WKH test: P |
| --- | --- | --- | --- | --- | --- | --- | --- | --- |
| Combined data tree | 198186.08157 | (best) | 0.000* | 0.000* | 0.000* | 0.000* | 0.000* | 0.000* |
| He (2008) ^b^ | 198913.94399 | 727.86242 | 0.000* | 0.000* | 0.000* | 0.000* | 0.000* | 0.000* |
| Wang (2007) ^a^ | 199281.37200 | 1095.29043 | 0.000* | 0.000* | 0.000* | 0.000* | 0.000* | 0.000* |
| CO1 gene tree ^a^ | 199561.02279 | 1374.94121 | 0.000* | 0.000* | 0.000* | 0.000* | 0.000* | 0.000* |

Associated probabilities are given and significantly (P<0.05) worse topologies are indicated by asterisks.

^b^ Bayes inference tree recovered from the dataset.

^a^ Maximum likelihood tree recovered from constraint search under the combined dataset.

Sequences of CO1 gene tree are retrieved from Genbank as well as unpublished data.
